# Nanoscale Electron Transfer Variations at Electrocatalyst–Electrolyte Interfaces Resolved by *in Situ* Conductive Atomic Force Microscopy

**DOI:** 10.1021/jacs.2c12617

**Published:** 2023-02-22

**Authors:** Martin Munz, Jeffrey Poon, Wiebke Frandsen, Beatriz Roldan Cuenya, Christopher S. Kley

**Affiliations:** † Helmholtz Young Investigator Group Nanoscale Operando CO_2_ Photo-Electrocatalysis, Helmholtz-Zentrum Berlin für Materialien und Energie GmbH, 14109 Berlin, Germany; ‡ Department of Interface Science, Fritz Haber Institute of the Max Planck Society, 14195 Berlin, Germany

## Abstract

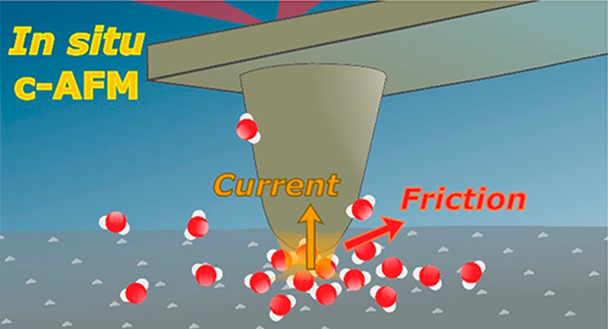

Rational innovation of electrocatalysts requires detailed knowledge of spatial property variations across the solid–electrolyte interface. We introduce correlative atomic force microscopy (AFM) to simultaneously probe, *in situ* and at the nanoscale, electrical conductivity, chemical-frictional, and morphological properties of a bimetallic copper–gold system for CO_2_ electroreduction. In air, water, and bicarbonate electrolyte, current–voltage curves reveal resistive CuO_*x*_ islands in line with local current contrasts, while frictional imaging indicates qualitative variations in the hydration layer molecular ordering upon change from water to electrolyte. Nanoscale current contrast on polycrystalline Au shows resistive grain boundaries and electrocatalytically passive adlayer regions. *In situ* conductive AFM imaging in water shows mesoscale regions of low current and reveals that reduced interfacial electric currents are accompanied by increased friction forces, thus indicating variations in the interfacial molecular ordering affected by the electrolyte composition and ionic species. These findings provide insights into how local electrochemical environments and adsorbed species affect interfacial charge transfer processes and support building *in situ* structure–property relationships in catalysis and energy conversion research.

## 1 Introduction

Local properties of electrode–electrolyte interfaces determine the overall performance of major catalytic reactions,^1^ including the electrocatalytic conversion of CO_2_,^2,3^ hydrogen,^4,5^ and oxygen,^6^ as well as battery-based electrochemical energy storage.^7,8^ Building knowledge-based design concepts for advanced (photo)electrocatalysts requires reliable structure–property relationships derived under liquid-phase reaction conditions.^9^ Associated with this fundamental quest are major challenges, in particular spatially mapping electric currents in photoelectrochemical systems,^5^ revealing interactions of anions and cations at electrochemical interfaces,^10,11^ resolving corrosion sites and material aging processes in electrochemical systems,^12^ or nanoscale probing of a material’s local catalytic activity and chemical nature of its reaction environment. Furthermore, insight into the interfacial ordering of water molecules and electrolyte ions with their effect on interfacial electron transfer (IET) needs to be gained, to rationally choose electrolytes and optimize electrochemical microenvironments.

In this context, correlative *in situ* analysis becomes critical to explore the relationships between structural, electric, and electrochemical properties at length scales commensurate with characteristic interfacial features,^13−19^ typically in the submicron to nanometer range. Such features can include surface reconstructions and changes in the electrode’s surface morphology or oxidation state.^20^ Owing to its capability of imaging local variations in the electric conductivity of surfaces, conductive atomic force microscopy (c-AFM)^21−26^ represents a promising contact-mode technique for *in situ* correlative imaging. The lateral resolution of current imaging in contact mode is determined by the tip–sample contact area, which is a function of the tip radius, load, local adhesion force, and surface deformation.^27,28^ While the majority of c-AFM studies were carried out in air^22,26,29−31^ and in vacuum,^33,35^ certain nonpolar liquids were used to provide inert environments.^34^ However, c-AFM imaging in polar liquids pertaining to electrocatalysis has not been reported yet, especially not in combination with friction force imaging.

In this work, we investigate bimetallic Au–Cu and polycrystalline Au electrocatalysts for CO_2_ electroreduction (CO_2_RR) in relevant aqueous electrolytes and provide nanoscale insights into the correlation between local current and friction force that are susceptible to molecular hydration layer ordering effects at the catalyst–electrolyte–probe interface. Our *in situ* conductive AFM approach enables localized current measurements with concomitant height and friction force imaging, a combination not readily available with other electrochemical probe techniques.^18,35^ We elucidate spatial variations in the IET rates through analysis of the current measured on individual grains of polycrystalline Au catalysts. By revealing spatial variations in the IET alongside the friction force signal and the morphology, this work sets the basis for correlative analysis of activity variations across catalyst–electrolyte interfaces in relevant chemical environments.

## 2 Results and Discussion

**Figure 1:**
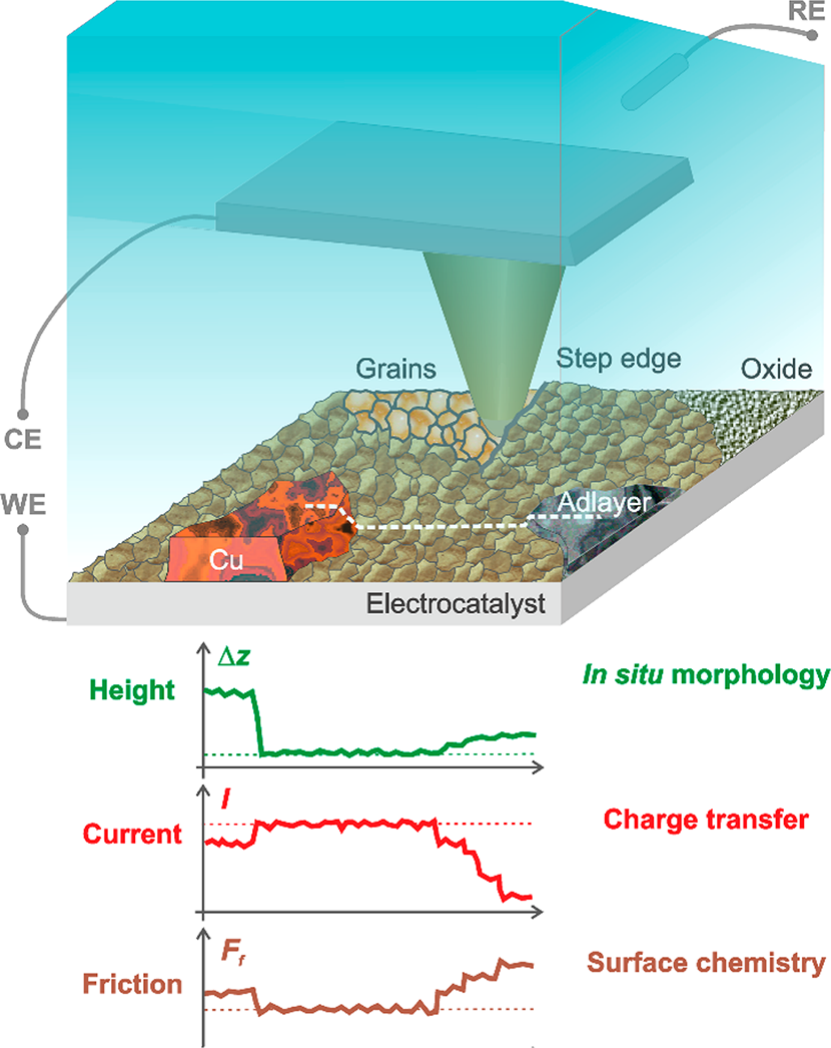
Schematic of an *in situ* correlative c-AFM setup for nanoscale characterization of electrified solid–liquid interfaces. The electrically conductive AFM cantilever is shown, together with simultaneous real-space imaging of an electrocatalyst’s local electrical, chemical-frictional, and morphological properties in an electrolyte and under potential control. Exemplary height, current, and friction traces, corresponding to the white dashed line. CE, RE, and WE denote the counter, reference, and working electrodes, respectively.

With the aim to probe spatial variations in the electric conductivity across the solid–liquid interface and to interrogate the interfacial layer of aqueous electrolyte, first we developed a setup for *in situ* imaging of current and friction. Figure 1 illustrates our contact mode AFM setup for simultaneous *in situ* imaging of an electrode’s morphology, the electric current across the tip–electrode contact, and the lateral force microscopy (LFM) contrast (for details on the setup see SI Sec. 1). The latter serves as a highly sensitive materials’ contrast and allows synergistic interrogation of mechanical and chemical heterogeneities,^36−38^ such as frictional analysis of *in situ* grown adsorption layers under electrochemical conditions.^39−41^ High measurement stability and inherent capability to continuously map large surface areas is achieved by employing solid Pt probes and TiN coated probes, thus nearly eliminating the risk of tip conductivity loss by abrasion.

**Figure 2:**
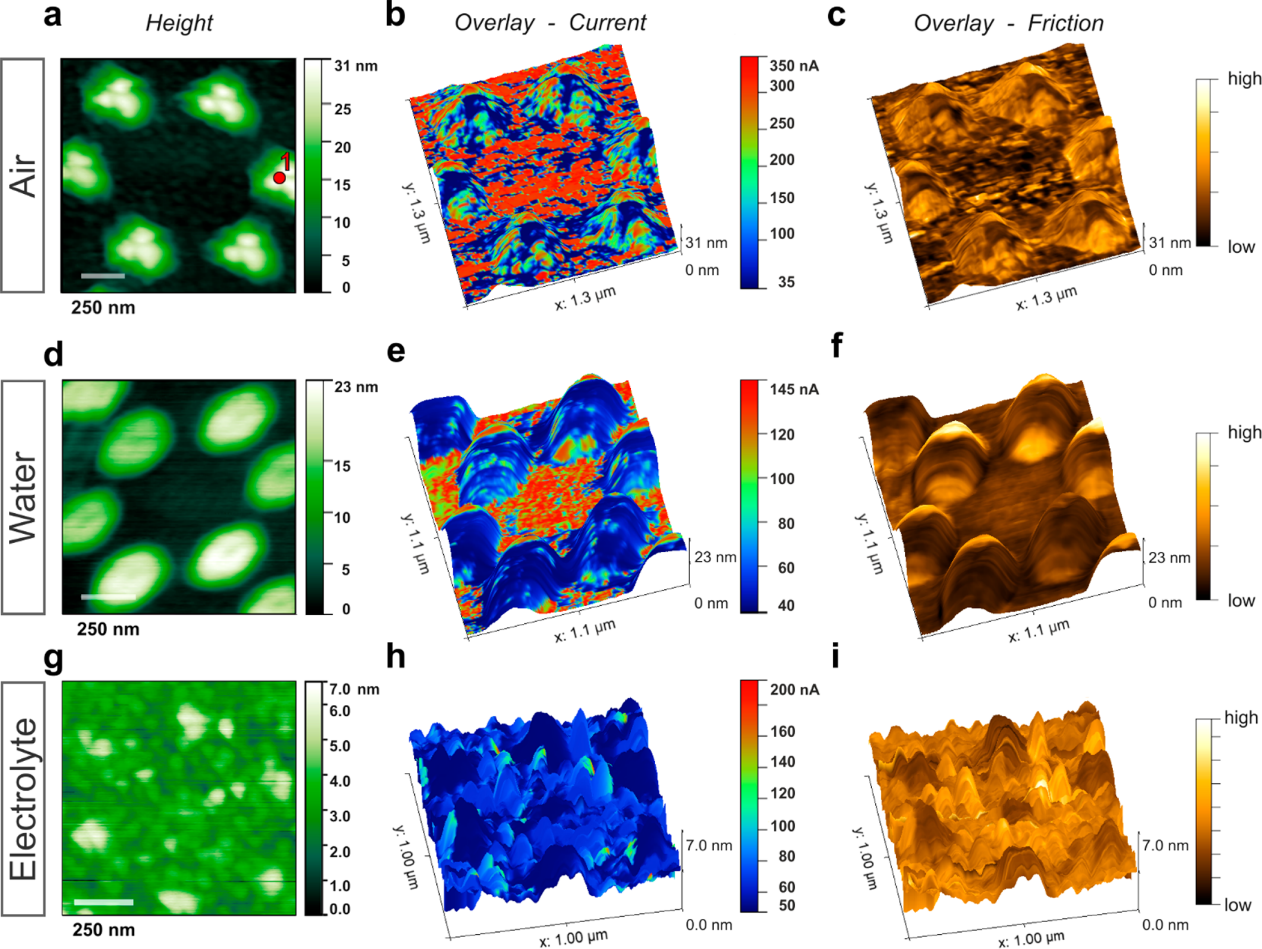
c-AFM imaging of bimetallic catalysts with Cu islands evaporated on Au, in air, water and potassium bicarbonate electrolyte. (a) Height image, where the CuO_*x*_ islands on the Au surface appear bright. Spot #1 marks the position where the *I–V* curves were measured on a CuO_*x*_ island. (b) Overlay of the current signal onto a 3D representation of the height image. The current was measured in air at *E*_*i*_ ≈ −289 mV. (c) Overlay of the friction force signal. (d–f) Height, current (*E*_*i*_ ≈ −130 mV vs Ag/AgCl) and friction force signals measured in water. (g–i) Height, current (*E*_*i*_ ≈ −597 mV vs Ag/AgCl) and friction force signals measured in aqueous 100 mM KHCO_3_ electrolyte. Solid Pt probes were employed, and absolute current values are given.

Next, we investigate bimetallic CO_2_RR electrocatalysts and demonstrate spatial mapping of electrical conductivity variations in air, water, and aqueous bicarbonate electrolytes. Figure 2a shows the AFM height image of a two-dimensional array of triangular Cu nanoislands patterned by nanosphere lithography (NSL)^42^ onto a polycrystalline Au film in air. Such regular arrays of Cu islands on Au or Ag emerged as tandem catalysts,^43^ specifically for CO_2_RR.^44,45^ A detailed analysis of the Cu–Au electrocatalyst by scanning electron microscopy (SEM), energy-dispersive X-ray (EDX) spectroscopy, and *ex situ* X-ray photoelectron spectroscopy (XPS) can be found in SI Secs. 2 and 3. The Cu islands are covered by a native oxide layer (Figure S3) and will be referred to as CuO_*x*_ islands, regardless of their particular surface chemical state.

**Figure 3:**
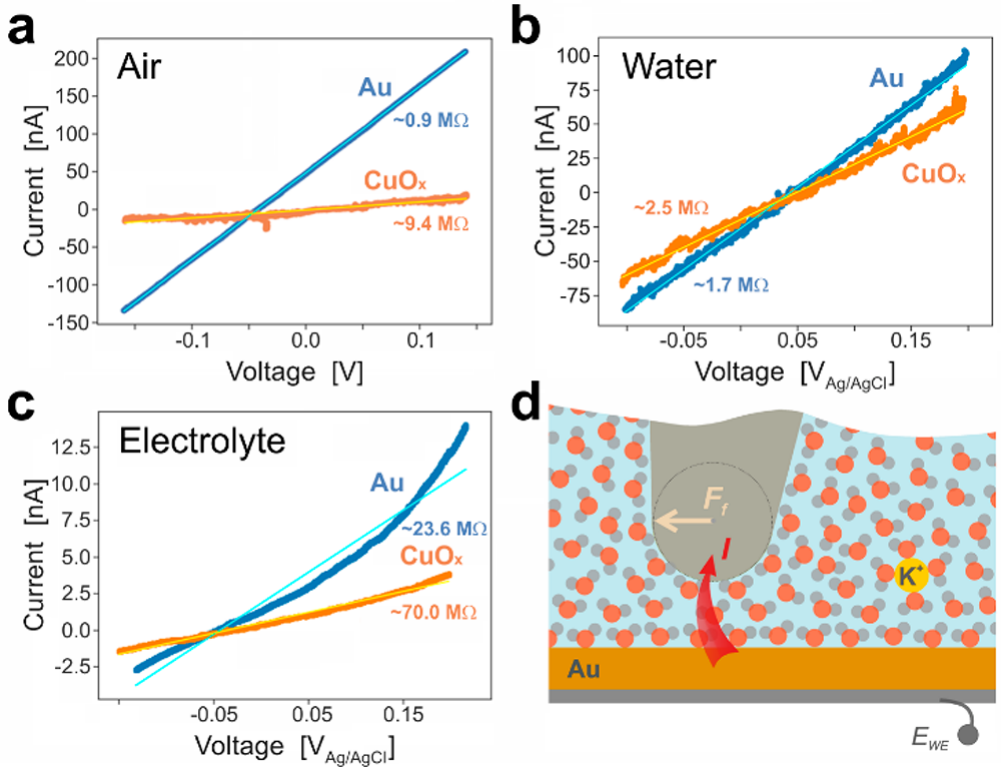
(a) Representative *I–V* curves measured, in air, on Spot #1 (on CuO_*x*_, as marked in Figure 2a, orange) and on Au (blue). Results of linear fits to the data are given by solid lines (in *orange* and *cyan*, respectively). Representative *I–V* curves measured on CuO_*x*_ and Au, in water are shown in (b) and in 100 mM KHCO_3_ electrolyte in (c). (d) Depiction of the water molecule ordering, for the case *E*_WE_ > 0, in the vicinity of the catalyst and tip surfaces, as well as a K^+^ cation (in *yellow*). A friction force *F*_*f*_ is acting on the tip scanning from left to right.

Figure 2b shows the height image (Figure 2a) overlaid with the corresponding current contrast in air. A lower electric current was measured on the CuO_*x*_ islands compared to the Au surface, in line with local *I–V* curves (Figure 3a) featuring a lower slope on CuO_*x*_ (Spot#1, as marked in Figure 2a) compared to Au. The box plot of the measured resistances (Figure S4a) shows that the values (and their variability) relating to a CuO_*x*_ island were larger than on Au, with mean values of ∼10.1 MΩ and 0.89 MΩ, respectively. Application of eq S3 (see SI Sec. 5) yields mean tip–sample contact resistance, *R*_*ts*_, values of *R*_*ts*__,Cu_ ≈ 10.2 ± 3.7 MΩ for the CuO_*x*_ island and *R*_*ts*__,Au_ ≈ 0.14 ± 0.01 MΩ for the Au surface. Linear *I–V* curves indicate ohmic behavior, as expected for contacts involving highly conductive metals (Au, Cu, Pt), while nonlinear *I–V* behavior could occur for a metal–insulator–metal configuration^30,31^ with a tunneling current across the tip–sample contact.

While the CuO_*x*_ surface layers are likely to make the predominant contribution to the overall resistance, electron scattering at the Au–Cu interface can also play a role. Interestingly, the *I–V* curves (Figure 3a) measured on CuO_*x*_ islands in air feature a much higher noise level than on Au (∼0.7 vs 0.1 nA), which could originate from different species present in the natural Cu oxide layers as well as from electronic effects,^46,47^ and be employed to locally distinguish different materials. As compared to the current contrast (Figure 2b), the friction force overlay shown in Figure 2c reveals a higher signal level on the CuO_*x*_ islands relative to the Au surface, as will be discussed later.

Figure 2e–f demonstrate first co-imaging of current and friction contrasts in ultrapure water. In agreement with the data obtained in air (Figures 2b–c and S6), the measured current was larger on the Au surface relative to the CuO_*x*_ islands (*I*^Au^/*I*^Cu^ > 1, Figure S4b), while the friction force was lower on the Au (*F*_*f*_^Au^/*F*_*f*_^Cu^ < 1, Figure S4c). We attribute the increased contact resistance to potential-induced interfacial water dipole layers^48^ that act as a barrier, which free electrons need to surpass for transferring from the electrode to the sensing AFM tip.^49^

The linearity of *I–V* curves (Figures 3a,b), as measured in air and water by solid Pt AFM probes, is consistent with the notion that for Pt surfaces the first water layer shows a metal-like electric conductivity, as a result of charge transfer between the water 3s and Pt 5d electrons.^50,51^ By increasing the applied voltage, both the signal-to-noise ratio can be enhanced (Figure S7) and a wider range of electrochemical interactions becomes accessible.

In aqueous 100 mM KHCO_3_ electrolyte, the current contrast appears relatively weak but qualitatively unchanged (Figure 2h). The average resistance values (Table ST1 in Sec. 8 of the SI) obtained from *I–V* curves (Figures 3c) were *R*_*ts*__,Au_ ≈ 30.0 ± 4.2 MΩ and *R*_*ts*__,Cu_ ≈ 57.0 ± 43.6 MΩ, i.e., significantly larger than the corresponding values obtained in air (*R*_*ts*__,Au_ ≈ 0.14 ± 0.01 MΩ and *R*_*ts*__,Cu_ ≈ 10.2 ± 3.7 MΩ, Figure 3a) and in water (*R*_*ts*__,Au_ ≈ 1.63 ± 0.58 MΩ and *R*_*ts*__,Cu_ ≈ 4.6 ± 7.6 MΩ, Figures 3b, S8). While the contact resistance shows a mild dependence on the load applied (here, cantilever-to-cantilever variations in the range ∼120 to 226 nN), the corresponding resistance variations, in the range of ∼23% (assuming a spreading resistance relationship^27^), are relatively small compared to the observed variations between Au and CuO_*x*_ islands (Table ST1 in SI Sec. 8).

Interestingly, *I–V* curves measured in the electrolyte reveal a degree of nonlinearity (Figure 3c, Figure S9), which we assign to altered local electric fields induced by ions accumulated at the electrified interface (Figure 3d). The *I–V* characteristics could be further affected by ion-induced changes in the island oxidation state or by surface hydroxylation.^52^ Adsorption of anions^53^ on the catalyst surface will lead to a negative surface charge and, thus, to an increased resistance for the flow of electrons across the catalyst/electrolyte/probe interface. The contact electric resistance method, where the electric current across macroscopic metal–electrolyte contacts is measured, was found to show large resistance variations for the case of anionic adsorbates, particularly from halide-containing electrolytes in the presence of gold, silver or copper.^54^ In qualitative terms, the configuration can be described by a triode, with the grid voltage related to the surface charge originating from ion adsorption and causing a current drop if the surface charge is negative.^54^ Notably, while in air and water the friction force contrast is higher on the CuO_*x*_ islands than on the surrounding Au (Figures 2c,f), this trend is reverted in the electrolyte (Figures 2i and S4c). We attribute the latter to ion-induced alteration of the local hydration layer ordering.

Specifically, under ambient or liquid conditions, the tip–sample contact comprises an interfacial hydration layer^55^ between the electrode surface and the Pt tip. When imaging in air, the capillary force acts as an additional normal force component,^56^ with a higher capillary force expected on Au that tends to be more hydrophilic than Cu (with respective water contact angles of ∼76° and 94°).^57^ As the friction force was larger on the CuO_*x*_ islands (Figure 2c), it seems unlikely that the contrast was dominated by the capillary force variations.

Upon fully immersing the AFM cantilever in water, the capillary force is eliminated. As can be seen from Figures 2f and S4c, qualitatively the same friction force contrast was observed as in air. Both in water and in the electrolyte, the tip–sample force interactions are affected by hydration effects.^28,58^ For instance, on hydrophobic areas the lateral force needed for water molecule displacement is likely to be reduced due to the interfacial depletion of water molecules.^38,59^ Interestingly, inverted friction force contrast occurred in the electrolyte (Figures 2i and S4c). The observed contrast reversal suggests that the presence of ions affected the interfacial hydration layers between the AFM tip and the catalyst surface. In terms of the electric double layer, electrolyte ions could be situated in the region between the Stern layers of the tip and the sample surface (Figure 3d) or adsorbed specifically at one or both interfaces of the tip–sample contact. Reportedly, specific ion adsorption can result in increased friction compared to a scenario of hydrated but mobile ions.^39^ While chaotropic, i.e. structure breaking, ions, such as Cs^+^, tend to reduce the level of hydration layer ordering, kosmotropic ions, such as Li^+^ and Na^+^, have the opposite effect.^60,61^ In turn, an increased ordering of water molecules caused by kosmotropic ions would result in a higher viscosity (SI Sec. 9) of the interfacial liquid and, thus, in a higher friction force counteracting the sliding motion of the AFM tip across the catalyst surface. Notably, the effect of kosmotropic ions has been elucidated in terms of strengthening of the hydrogen bond network of water.^62^ In this context, the friction force contrast of Figure 2i suggests that the kosmotropic effect was more pronounced on Au than on the CuO_*x*_ islands. Looking at its position in the alkali metal group, it seems unlikely that K^+^ ions (Figure 3d) show a distinct chao- or kosmotropic behavior (SI Sec. 9). Beyond K^+^ cations, Cu cations, originating from the CuO_*x*_ reduction and dissolution process occurring in the electrolyte (Figure S10) and adsorbing onto the Au surface, could lead to an increase in friction force levels.^40^

The above results for the class of bimetallic electrocatalysts demonstrate that electric conductivity variations across nanopatterned CuO_*x*_ islands on Au electrodes can be spatially resolved (Figure 2), despite the likely occurrence of leak currents (Figure S11), which can impair both the spatial resolution and the current sensitivity. Importantly, these results raise the question how the hydration layer impacts interfacial charge transfer. To this end, we investigated a polycrystalline gold electrode at granular length scale.

**Figure 4:**
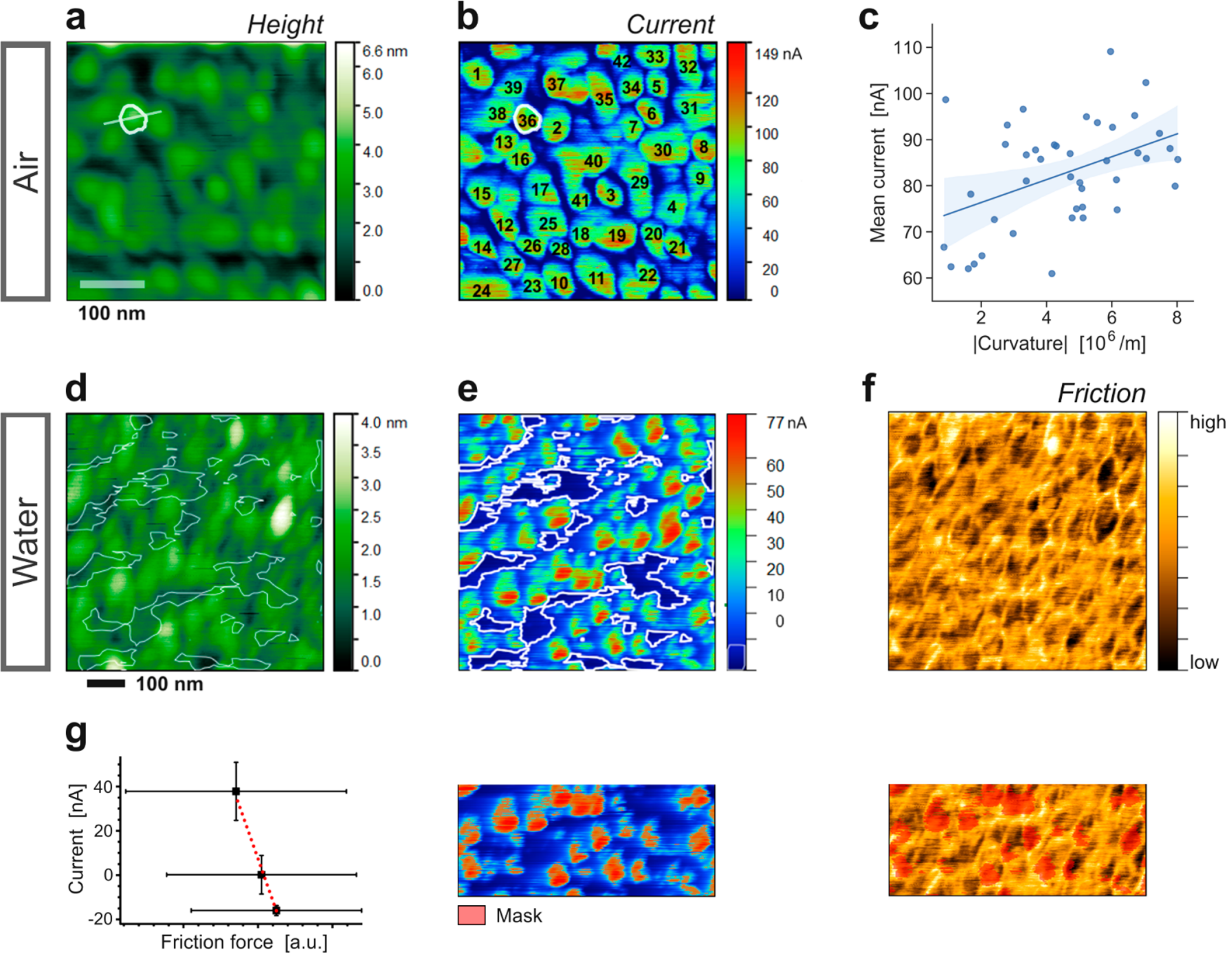
c-AFM imaging of a polycrystalline Au electrocatalyst surface in air and in water. (a) Height image, recorded in air, with grain #36 and its cross-section for profile analysis marked with white lines. (b) Current image, measured in air at *E*_i_ ≈ +220 mV. (c) Plot of the mean current measured on individual grains versus the grain curvature. Solid blue line: linear trendline. (d) Height image of a polycrystalline Au surface, in water. (e) Current image, measured at *E*_i_ ≈ +340 mV vs Ag/AgCl. Thin white lines indicate low current regions. (f) Corresponding friction force image. (g) Panel illustrating the correlative analysis of current and friction force images by splitting the total current range into 3 regimes and evaluating the mean current and friction values for each regime. The resulting current vs friction plot is shown on the left. All images were obtained using TiN-coated tips.

Figure 4a shows the height image of a polycrystalline Au surface measured in air, with a Au grain size distribution in the range of 2*r*_eq_ ≈ 4 to 76 nm (Figure S12). Figure 4b reveals that currents on the convex-shaped grains (Figures 4a, S13a) were larger than on intergranular regions, indicating lower electric conductivity of near-surface grain boundary regions compared to intragranular regions. A current contrast reversal by effect of tip–sample contact area variations can be ruled out (Figure S14). This variation potentially originates from locally increased defect densities and enhanced free electron scattering at grain boundaries, in accordance with an electron mean free path of ∼37 nm (at 300 K for Au^63^) commensurate with the measured grain size distribution (Figure S12). An example of mesoscale defects, such as microcracks, which can lead to electrically insulated grains, is given in Figure S15.

Notably, analysis of individual grains (Figures 4a–b, S13a–b) yields a positive current-versus-curvature trend (Figure 4c, Pearson correlation coefficient, PCC, of ∼0.435, medium strength^64^), indicating curvature-related local electric field enhancement. Effective grain shape parametrization was accomplished by parabolic fits yielding very good agreement with measured grain profiles (Figure S13a), whereas the corresponding cross-sectional current image profile was described by the mean current measured on the grain (Figure S13b). The electric field enhancement effect occurs in the vicinity of highly curved metal nanostructures, including Au nanocones,^65^ and was reported to boost the CO_2_RR kinetics of nanoneedle arrays^14,15^ via field-induced concentration of cations from the electrolyte. Further analysis (Figure S13c) showed that the mean current increased mildly with the maximum grain height (PCC ≈ 0.470). The complementary plot of grain curvature vs maximum height (Figure S13d) suggests that grains with higher peaks are more curved (PCC ≈ 0.576). In terms of AFM imaging, elevated grains can be more accurately resolved than recessed ones, particularly in the extreme case of a relatively blunt tip prone to the dilation effect^66^ when tracing highly curved features (Figure S16c).

Figure 4d–f present c-AFM imaging results obtained in water. The Au grains were found to carry larger currents compared to intergranular regions (Figures 4e, indicated by lines). A correlative analysis of the height and current images of Figure 4d–e alongside the corresponding friction force image (Figure 4f) is presented in Figure S17 and illustrated in Figure 4g. While the mean friction force slightly decreased with increasing mean height (Figure S17d), the mean current was found to increase. Overall, the analysis suggests that the current was higher where the friction force was lower (Figures S17e, 4g). Such a trend may shed light onto the interfacial arrangement of water molecules and associated ions, further to the correlations discussed in conjunction with contrasts measured in air (Figure 4a–c). The anticipated variations of friction force and tip–sample current can be rationalized in terms of the electrolyte local viscosity. While an increase in the latter (SI Sec. S9), reflecting interfacial ordering of water molecules and dissolved ions, results in a higher friction force, the current should decrease if the interfacial ordering entails a higher energetic barrier for the transfer of electrons. Consequently, current images appear darker and friction force images brighter (Figure S17a–c) in regions of high interfacial ordering. Indeed, results from shear force spectroscopy showed that higher friction forces occur for the case of ordered water layers governed by a hydrogen bonding network, whereas a lubricating effect can result from low concentrations of ions disturbing the regular arrangement of water molecules.^67^

In the model developed by Liu et al.,^49^ the potential barrier for electron charge transfer is assigned to a single layer of water dipoles aligned in the electric field (Figure S18). Considering the scenario of the tip–sample contact (Figure 3d) that highlights the hydration layers of both sample and tip (with a fraction of the interfacial water molecules not being perfectly aligned with the electric field), however, the interfacial water is likely to encompass several layers with various degrees of molecular order. Hence, the width and the height of the energy barrier that the electrons need to traverse will be a function of the hydration layer ordering. In particular, the chao- or kosmotropic effect of electrolyte ions is likely to affect the potential barrier in its height or width or shape. In fact, the electron transfer kinetics will be impeded if the energy penalty for the water molecular rearrangement needed for electron transfer is too high, as shown for the case of the ferro/ferricyanide redox system [Fe(CN)_6_]^3-/4–^.^68,69^ Notably, more disordered solvation environments at the electrolyte/electrode interface can increase the exchange current density and reduce the reorganization energy.^70,71^

Beyond such coupling between the friction force and the hydration layer structure, spatial friction force variations can also be indicative for interfacial adsorption processes or oxidative reactions. Reportedly, the adsorption of hydroxide anions onto Au(111) surfaces in a perchloric electrolyte entails an increase in friction force, and at higher anodic potentials such adsorption can lead to surface oxidation.^72^ Considering that the negative surface charge associated with adsorbed hydroxide anions raises the potential barrier (Figure S18) for IET, such adsorption processes are likely to modulate the electron transfer rates.

To investigate how the presence of adlayers can affect the IET, we carried out *in situ* c-AFM imaging in mixed electrolytes pertaining to CO_2_RR. Recently, electrolytes containing halide ions were found to lower the overpotential and improve the CO_2_RR performance, by anion adsorption and corresponding negative surface charges.^73,74^ Similarly to observations in water, Figure S19d–f reveal relatively low currents measured over concave intergranular regions of a polycrystalline Au surface in a binary electrolyte of CO_2_-saturated 100 mM KHCO_3_ mixed with 50 mM KCl. While most grains appeared bright in the current image obtained in air (Figure S19b), in the mixed electrolyte the number of grains with clear current contrast was diminished (Figure S19e). Consistently with the likely presence of a negatively charged adlayer of Cl^–^ ions adsorbed under the *in situ* c-AFM imaging condition, the current level was relatively low throughout an extended region, most likely by suppressing the transfer of electrons due to the higher energetic barrier associated with the negative surface charge.^54^ Moreover, *in situ* c-AFM allowed identification of patches of adsorbed species (Figure S20) that tend to reduce the electrocatalytically active area by diminishing or even blocking the interfacial electron flow. Such blocking layers can originate from electrolyte constituents, such as carbonaceous species, adsorbing over time.^75^

## 3 Conclusions and Outlook

We presented a novel *in situ* correlative microscopy approach that enables simultaneous real-space imaging of an electrocatalyst’s local electrical, chemical-frictional, and morphological properties in aqueous media and under potential control. Allowing flexible choice of AFM tip materials, our *in situ* conductive AFM combined with lateral force microscopy is broadly applicable to the nanoscale characterization of electrified solid–liquid interfaces.

First, for bimetallic CO_2_RR electrocatalysts, we spatially resolved electric conductivity variations across nanopatterned CuO_*x*_ islands on Au electrodes in air, water, and bicarbonate aqueous electrolytes. Consistently with current contrasts that displayed catalyst surface areas of different composition, *in situ* measured current–voltage curves showed highly resistive CuO_*x*_ islands. Concomitant friction force images indicated a qualitative contrast variation upon change from water to a bicarbonate electrolyte, thus confirming the capability of gaining further insight into ordering effects in interfacial water layers. Second, for nanocrystalline Au electrocatalysts, we showed that convex intragranular Au regions appear more conductive than concave intergranular ones, with analysis of individual grains indicating a positive current-curvature correlation, presumably due to electric field enhancement. In water, the observation of interfacial regions of lower currents, accompanied by higher friction forces, was rationalized in terms of the degree of ordering in the interfacial liquid layers. Further to the heterogeneity of nanocrystalline Au, the interfacial electron transfer depends on the water molecular arrangement, interrogated concomitantly by friction force imaging.

Local current measurements under (photo)electrochemical conditions enable investigation of interfacial charge transfer processes, thus revealing catalyst heterogeneities, visualizing catalytically active/passive regions or detecting corrosion sites. Nonetheless, for accurate current measurement on semiconducting photocatalysts, the AFM tip needs to be specially engineered in a way that the tip apex is conductive but no tip–electrolyte–catalyst current flows through other portions of the tip surface. We envision that such advanced probes will serve to enhance our understanding of the fundamental properties of solid–liquid interfaces. This includes unveiling the ordering of hydration layers through friction force imaging as a function of the ion species and electrolyte concentration, but also gaining insight into a catalyst’s local wetting behavior. Beyond CO_2_RR, the presented approach could feed into analyses of various key electrocatalytic reactions and (photo)electrochemical interfaces, with applications relating to electrochemical energy storage systems, chemical sensing, or corrosion science.

## Materials and Methods

### Materials

For preparation of K_2_CO_3_ aqueous electrolytes, puratronic grade potassium carbonate salt of 99.997% purity (metals basis) from Alfa Aesar was mixed with HPLC LC-MS grade ultrapure water from VWR. For saturation with CO_2_, the K_2_CO_3_ aqueous electrolytes were purged with the gas for ∼30 min. A Si wafer (n-type doping, electric resistivity ∼15 ± 3 MΩ cm) with a 50 nm Au thin film on top of a 70 nm Ti film was obtained from MicroFabSolutions; the wafer chip size was ∼12 × 12 mm^2^. A polished Au polycrystal of 1.7 mm thickness was supplied by Surface Preparation Laboratory. A monodisperse 757 ± 19 nm polystyrene sphere suspension of 5%/wt was obtained from MicroParticles GmbH.

### Nanosphere Lithography

Controlled placement of copper sites was achieved through NSL, whose process details are outlined in the literature.^42^ In short, a 300 μL monodispersed polystyrene sphere suspension was added to 300 μL 1%/vol styrene solution in ethanol and 10 μL of 0.1%/vol sulfuric acid. This mixture was gently dosed onto the surface of a Petri dish of ultrapure water (18.2 MΩ cm resistivity) through a curved glass pipet for the particles to self-assemble into a hexagonal close packed layer, with centimeter-scale domain formations and clear iridescence, covering approximately 80% of the water surface. The layer was consolidated by adding the dilute surfactant, and Au-coated (∼50 nm thickness) Si wafer substrates were placed underneath the assembled layer into the water. The water was gently drained using a siphoning tube, placing the layer onto the substrate and left to dry. Position-controlled Cu site placement was achieved by electron beam deposition of 30 nm Cu onto the sphere-coated Au-covered Si substrates. The polystyrene spheres were subsequently removed by 30 s sonication in ultrapure water and rinsed with more water. The substrate was then blow-dried, using compressed air.

### Atomic Force Microscopy

A Cypher ES AFM system (by Asylum Research/Oxford Instruments), fitted with an environmental scanner for imaging in liquid, was employed for the c-AFM measurements. The probe was mounted onto an EC AFM type cantilever holder using a clip. The clip was made of poly(ether–ether–ketone) (PEEK) and served as a connector between the probe and the EC cell core component made of quartz glass. Nearby the clip, the free end of a Ag wire was held in place and employed as a quasi-reference electrode (QRE) when measuring in liquid. When measuring in air, the potentiostat was operated in two-electrode configuration, which was also used for control measurements in water (Figure S8). For electric connection, a flat flexible cable was attached to the chip-sided end of the probe using silver conductive epoxy (circuitworks CW2400 by Chemtronics). Via a zero-insertion force (ZIF) connector mounted onto a miniature PCB and a coaxial cable with SMB connectors, the flat flexible cable was connected to the counter electrode (CE) terminal of the ultralow current (ULC) unit connected to the potentiostat (SP-200 by BioLogic). Correspondingly, the sample back-electrode was connected to the working electrode (WE) terminal. The sample was mounted onto a liquid cup assembly fitted with a perfluoroelastomer O-ring. To prevent mechanical contact between the sample surface and the contact point between chip and the flat flexible cable, the cantilever/chip was mounted at a slightly steeper inclination angle. Typically, the sum signal was ∼4 V, or higher, and sufficient for robust feedback performance in contact mode.

AFM contact mode cantilevers with an electrically conductive TiN coating were used as well as solid Pt probes. The probes of type TiN-FORTA (by Applied Nanostructures, CA) had an average spring constant of ∼3.8 ± 0.3 N/m, a nominal tip height of ∼15 μm, and featured a tip-sided TiN coating with a nominal thickness of ∼50 nm. The nominal radius of curvature of the TiN coated AFM tips was ∼30 nm. Furthermore, conductive probes of type 25Pt400B (by Rocky Mountain Nanotechnology) were used that feature a solid Pt tip. Their average spring constant, nominal tip shaft length, and tip radius were ∼6.2 ± 2.9 N/m, ∼80 μm, and <20 nm, respectively.

Control and data acquisition of the voltages applied and the currents measured were through the SP-200 potentiostat and the EC-Lab control software (by Bio-Logic Science Instruments). To minimize the risk of noise coupling, the ULC unit was mounted next to the Cypher enclosure and shielded electromagnetically. For c-AFM imaging, a chronoamperometry (CA) routine was run, which allowed setting of the applied potential and recording of the measured current over time. Corresponding analog signals available from the IS1 isolation module (by Bio-Logic) were fed into the Cypher controller ARC2. In this way, voltage and current images were recorded simultaneously to the deflection, height, and lateral force images pertaining to AFM contact mode. To account for the time constant of the potentiostat (typical current range setting ∼1 μA), the scan rate was in the range of ∼0.15 to 0.25 lps for an AFM image of 256^2^ pixels.

For measurement of *I*–*V* curves, the applied voltage was ramped via the linear sweep voltammetry (LSV) routine of the potentiostat, typically over a range between −650 and +480 mV vs Ag. These values can be converted to RHE potentials *E*_RHE_, according to *E*_RHE_ = *E*_Ag/AgCl_ + 0.235 V + 0.059 V × pH, with *E*_Ag/AgCl_ ≈ *E*_Ag_ + 0.05 V. To eliminate the contribution from transient (capacitive) currents, prior to the voltage sweep a CA was run at the sweep starting voltage. Depending on the decay time of the transient currents, the duration of this initial CA was in the range 20 to 100 s. To minimize capacitive currents occurring in the course of the voltage sweep, the sweep rate was set to a relatively low value in the order of 1 mV/s. The resistance d*V*/d*I* was obtained from linear regression applied to the *I*–*V* curves. From the thus obtained overall resistance, the tip–sample contact resistance, *R*_*ts*_, was calculated by accounting for the serial and parallel resistances included with the front-end electric circuit (Figure S5 and eq S3). The electric resistance between sample back-electrode and Au surface was in the ∼1 kΩ range and, thus, negligible compared to the MΩ range *R*_*ts*_ values.

The software package Gwyddion version 2.55 (by Czech Metrology Institute) was employed for image processing of the AFM images, including the preparation of overlays. Current images were exported from the AR/OI control software to ASCII format, then converted from mV to nA, and finally imported to Gwyddion. Friction force images were obtained from the LFM images recorded in trace and in retrace by calculating 0.5 times the difference between these images, a common procedure for the elimination of lateral force components originating from surface slope variations.^76^ Python 3.7.6 under the IDE Spyder 4.1.2 was employed for general data analysis purposes and the preparation of plots.

### Scanning Electron Microscopy

For SEM imaging, a Hitachi S-4800 system including a cold field emission gun was employed, allowing for acceleration voltages in the range from 0.1 to 30 kV. In addition to separate detectors for backscattered, secondary, and transmitted electrons, it was fitted with a Quantax 800 (by Bruker) unit, including Xflash 6 for EDX analysis.

### X-ray Photoelectron Spectroscopy

XPS analysis was carried out *ex situ*, 24 h after preparation of the NSL samples, with the samples stored in a desiccator purged with N_2_ gas. XPS analyses were performed in an ultrahigh-vacuum instrument, at a base pressure below 5 × 10^–9^ mbar, with an Al X-ray source (1486.6 eV, 300 W, XR 50; by Specs GmbH) and a hemispherical electron analyzer (Phoibos 100, by Specs). The emission normal (90°) to the surface was analyzed. A pass energy of 100 eV was selected for all survey scans and 30 eV for all regional high resolution spectra. The Au 4f peak at 84.0 eV was used as an internal reference for the binding energy scale. Detailed analysis of regional peaks was conducted using CasaXPS (by Casa Software, UK). Most peak fittings used 70% Gaussian and 30% Lorentzian (GL(30) setting) peaks, and Cu LMM spectra were fitted with reference Cu, Cu_2_O, CuO line shapes.

## Supporting Information Available

A schematic of the *in situ* c-AFM experimental setup; AFM imaging and EDX analysis of an NSL sample; XPS analysis of an NSL sample; summary of results from c-AFM *I*–*V* curves, in air, on the Cu–Au bimetallic catalyst (Figure 2); relationship between the measured resistance and the tip–sample resistance; complementary example of c-AFM and friction force imaging of an NSL sample, in air; *I–V* and noise characteristics for the case of polycrystalline Au surfaces in water; c-AFM based *I–V* curve measurements of an NSL sample, in the electrolyte; *B*-coefficient of the Jones–Dole relationship between electrolyte concentration and viscosity; copper island height variations upon exposure to water and 100 mM KHCO_3_ aq. electrolyte; description of the relative contribution of potentially occurring leak currents; grain size distribution of the polycrystalline Au film; curvature and mean current of individual gold film grains; single observation of a reverted c-AFM current contrast between granular and intergranular regions; locally reduced current associated with a single grain; effect of the finite tip size on the AFM imaging of a polycrystalline morphology; correlative analysis of height, current, and friction force images of a polycrystalline Au electrocatalyst surface in water; electron energy scheme for the interfacial electron transfer from a metal electrode; c-AFM and friction force contrasts of a polycrystalline Au surface, in air and in a mixed electrolyte; electrically blocking patch on top of a Au polycrystalline film (PDF)
