# Supplementary material for: Nanoscale Electron Transfer Variations at Electrocatalyst–Electrolyte Interfaces Resolved by in Situ Conductive Atomic Force Microscopy
Source: J Am Chem Soc. 2023 Feb 22;145(9):5242–51. doi: 10.1021/jacs.2c12617 (PMC9999420; doi:10.1021/jacs.2c12617)
Supplement: Supplementary file 1 — ja2c12617_si_001.pdf [file ja2c12617_si_001.pdf]

Supporting Information for

Nanoscale electron transfer variations at electrocatalyst-electrolyte interfaces resolved by *in situ* conductive atomic force microscopy

Martin Munz,[1,2] Jeffrey Poon,[2] Wiebke Frandsen,[2] Beatriz Roldan Cuenya,[2] and Christopher S. Kley [1,2] *

[1] Helmholtz Young Investigator Group Nanoscale Operando CO_2 Photo-Electrocatalysis, Helmholtz-Zentrum Berlin für Materialien und Energie GmbH, 14109 Berlin, Germany

[2] Department of Interface Science, Fritz Haber Institute of the Max Planck Society, 14195 Berlin, Germany

* *Corresponding author.* Email: christopher.kley@helmholtz-berlin.de, kley@fhi-berlin.mpg.de

Contents

1) Schematic of the *in situ* c-AFM experimental setup

2) AFM imaging and EDX analysis of an NSL sample

3) XPS analysis of an NSL sample

4) Summary of results from c-AFM *I-V* curves, in air, on the Cu-Au bimetallic catalyst (Figure 2)

5) Relationship between the measured resistance and the tip-sample resistance

6) Complementary example of c-AFM and friction force imaging of an NSL sample, in air

7) *I-V* and noise characteristics for the case of polycrystalline Au surfaces in water

8) c-AFM based *I-V* curve measurements on an NSL sample, in the electrolyte

9) *B*-coefficient of the Jones-Dole relationship between electrolyte concentration and viscosity

10) Copper island height variations upon exposure to water and 100 mM $KHCO_3$ aq. electrolyte

11) Description of the relative contribution of potentially occurring leak currents

12) Grain size distribution of the polycrystalline gold film

13) Curvature and mean current of individual gold film grains

14) Single observation of a reverted c-AFM current contrast between granular and intergranular regions

15) Locally-reduced current associated with a single grain

16) Effect of the finite tip size on the AFM imaging of a polycrystalline morphology

17) Correlative analysis of height, current and friction force images of a polycrystalline Au electrocatalyst surface in water

18) Electron energy scheme for the interfacial electron transfer from a metal electrode

19) c-AFM and friction force contrasts of a polycrystalline Au surface, in air and in a mixed electrolyte

20) Electrically blocking patch on top of a Au polycrystalline film

Supplementary references

1) Schematic of the *in situ* c-AFM experimental setup

A prerequisite for *in situ* AFM imaging is the integration of an electrochemical (EC) cell with the AFM setup including the AFM probe, the AFM scanner and a laser beam for cantilever deflection detection as the core elements. Here, the EC cell liquid volume was confined by the sample surface, a cell body and a glass ceiling (Fig. S1). An elastomer O-ring was clamped between the sample surface and the cell body, to prevent leakage of the electrolyte.

Subsequently to imaging a region of interest in c-AFM mode, *I-V* curves can be recorded on selected surface motifs and under CO_2RR relevant conditions.

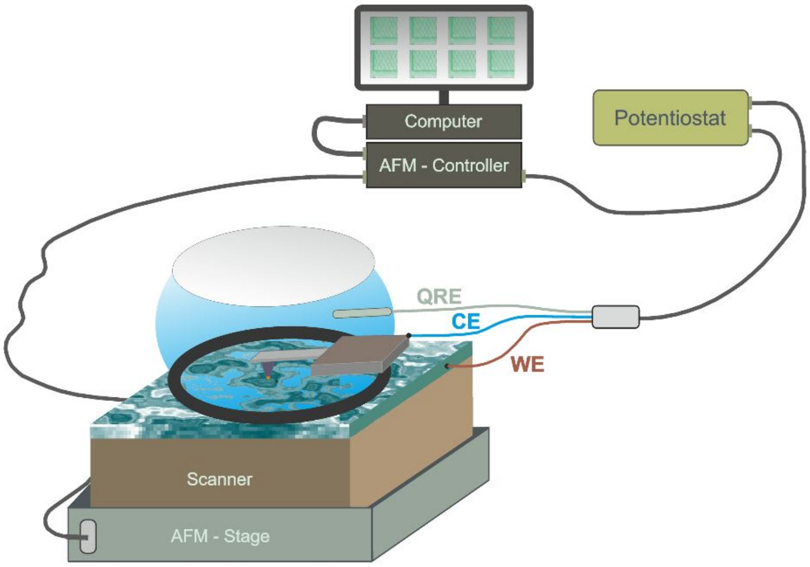

Figure S1. Schematic representation of the setup for *in situ* c-AFM. An electrically conductive AFM cantilever is mounted onto the EC cell (cell side walls *not shown*, for the sake of clarity) that is filled with an aqueous electrolyte of low ionic concentration. An elastomer O-ring acts as a seal between the sample surface and the liquid volume. A flat ceiling made of glass closes the cell and acts as a transparent window for the laser beam (*not shown*) sensing the deflection of the cantilever. The cantilever chip is connected to the counter electrode (CE) terminal of a potentiostat for measurement of the electric current across the tip-sample contact, while the sample is connected to the working electrode (WE) terminal. A silver wire serves as a quasi-reference electrode (QRE). However, the potential values reported in this study are referenced to Ag/AgCl, by adding an offset of ~50 mV (see Section MATERIALS AND METHODS – Atomic force microscopy). Further key components of the AFM setup, including controller, stage and scanner, are depicted schematically and not drawn to scale.

2) AFM imaging and EDX analysis of an NSL sample

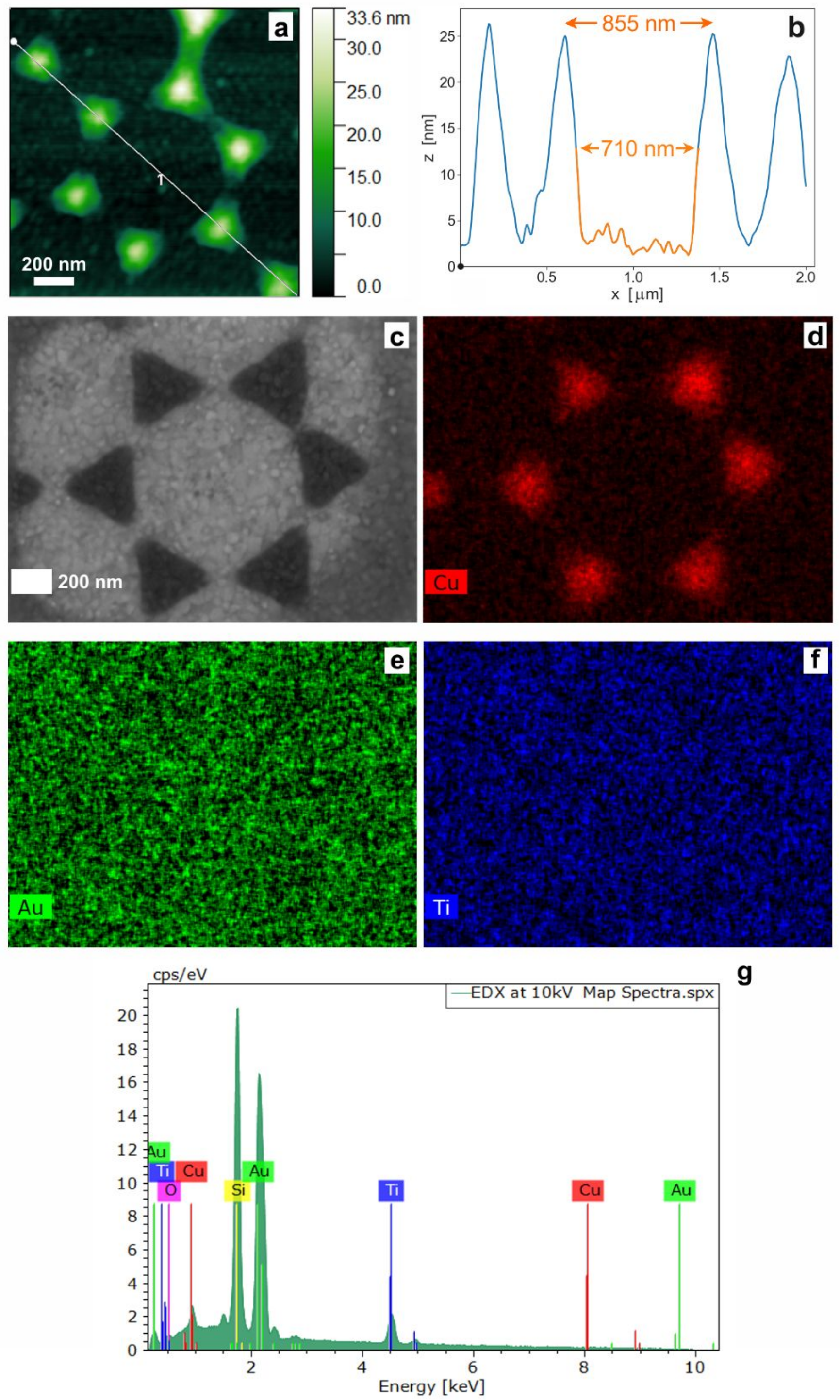

Figure S2. AFM imaging and EDX analysis of a patterned sample made by NSL. The pattern of Cu islands was supported by a Si wafer chip with a bilayer coating of Ti and Au. **a** AFM height image, measured in air. **b** The cross-sectional profile along the diagonal line marked in (a). **c** SEM micrograph of a single hexagonal array. **d** Corresponding EDX map for Cu, at a beam energy of 10 keV. **e** EDX map for Au. **f** EDX map for Ti. **g** EDX spectrum. Further to Au, Cu and Ti, the spectrum indicated the presence of Si and O.

As shown in Figs. S2a-b, the average Cu island edge length and maximum thickness were ~202 and ~24.7 nm, respectively. The distance between convex-shaped Cu islands was in the range of ~855 nm (peak-to-peak distance) to ~710 nm (distance measured at half peak height) (Fig. S2b), corresponding to the diameter of the latex spheres (~757 ± 19 nm) used for the self-assembly of the NSL mask. Chemical mapping by energy-dispersive X-ray (EDX) spectroscopy (Figs. 2, Fig. S2) confirmed that the islands (Fig. S2c) were made of Cu (Fig. S2d).

Corresponding to the Ti / Au bilayer chosen as a support surface for the Cu islands, non-zero intensities of both metals were detected across the entire surface (Figs. S2e-f). Additional to these metals, the EDX spectrum (Fig. S2g) indicates the presence of O and Si. Our average sampling depth (~200 nm at ~10 keV,), exceeds the total thickness of the Ti (~70 nm), Au (~50 nm) and Cu (~30 nm) layers, thus reaching into the Si substrate.

3) XPS analysis of an NSL sample

Ex situ X-ray photoelectron spectroscopy (XPS) analysis of the NSL samples (Fig. S3), revealing the presence of Cu(II) in the Cu 2p high-resolution spectrum (Cu(II) satellites in Fig. S3c). Our spectroscopic analysis indicated the abundance of cupric oxide (CuO, ~66%) as compared to cuprous oxide (Cu_2O, ~27%), in agreement with reports [1,2] stating that atmospherically-formed copper oxide layers contain Cu_2O or a CuO-on-Cu_2O bilayer. The presence of Cu(II) in the Cu 2p high-resolution spectrum is confirmed by the Cu(II) satellite that appears around ~943 eV (Fig. S3c).

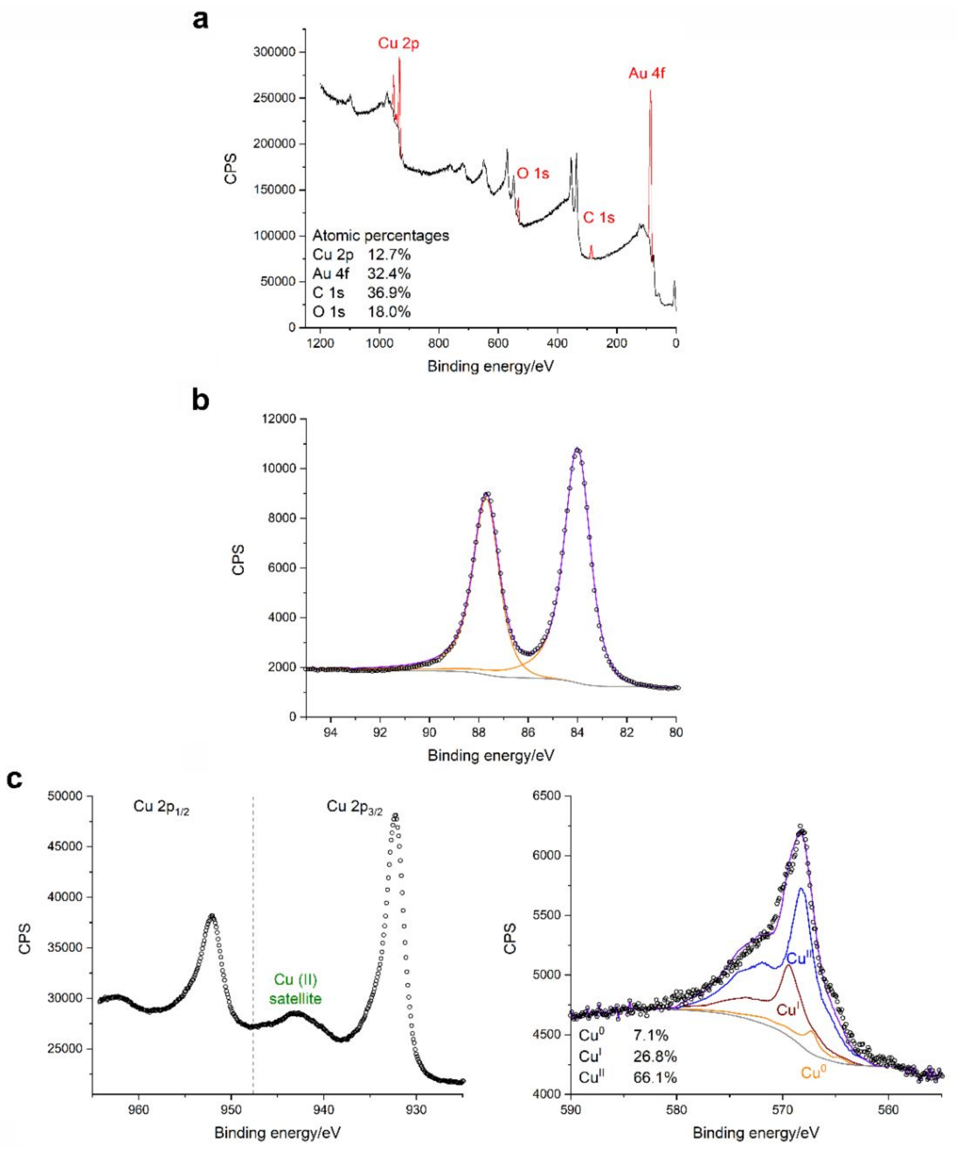

Figure S3. a Survey spectrum of a Cu nanosphere lithography patterned sample deposited on Au, recorded over the binding energy range up to 1200eV. **b** High-resolution spectrum of the Au 4f peaks. **c** High-resolution spectra of the Cu 2p (*left*) and Cu LMM peaks (*right*).

4) Summary of results from c-AFM *I-V* curves, in air, on the Cu-Au bimetallic catalyst (Figure 2)

It should be noted that the calculation of R_{ts} is based on the nominally applied potential. However, changes in the surface potential tend to be smaller than the changes in the applied potential, due to the gradient in potential between the electrode surface and the outer Helmholtz plane of the electric double layer (EDL) [3], [4]. Thus, the tip-sample resistance values based on surface potential changes would be smaller than the calculated R_{ts} values, given the same changes in the measured current.

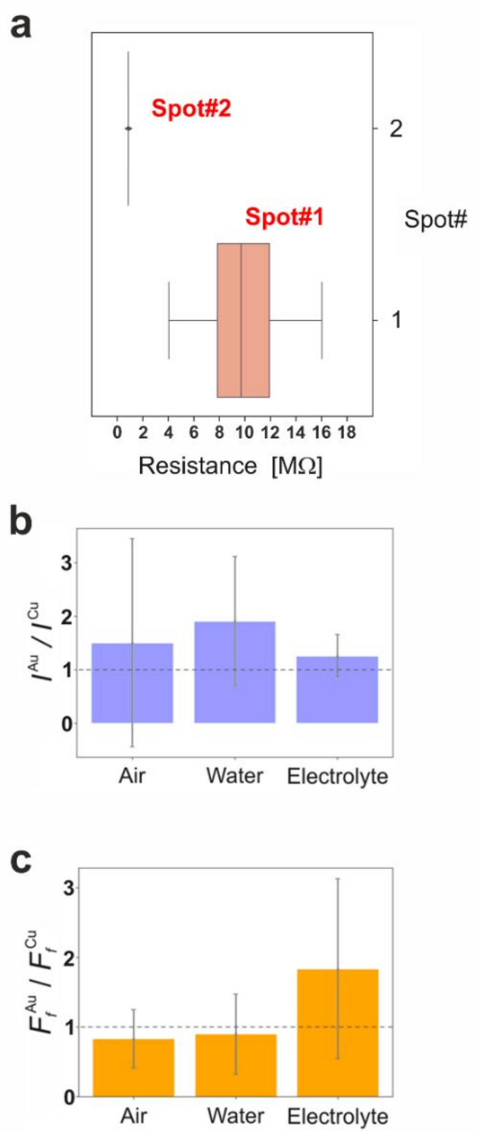

Figure S4. Summary of results from Fig. 2. a Box plot of the resistance values from both sets of *I-V* curves measured in air, i.e. curves measured on Au and on CuO_x. Application of Eq. (S3), as given in SI Sec. 5, yields mean tip-sample contact resistance, R_{ts}, values of $R_{ts,Cu}$~10.2 ± 3.7 MΩ ($R_{ts,min}$~3.4 MΩ, $R_{ts,max}$~17.2 MΩ) for the CuO_x island and $R_{ts,Au}$~0.14 ± 0.01 MΩ for the Au surface. **b** Bar plot of the current ratios I^{Au} / I^{Cu} related to the current contrasts in air, water and the electrolyte (Figs 2b, e, h). **c** Bar plot of the friction force ratios F_f^{Au}/F_f^{Cu} related to the contrasts of Figs. 2 c, f, i.

5) Relationship between the measured resistance and the tip-sample resistance

A relationship between the measured resistance, R_m, and the true resistance has been determined by measuring *I-V* curves for a range of dummy resistors, R_d.

In addition to the tip-sample contact resistance, the front-end electric circuit included a serial resistor, R_s, as well as a small resistance, R_l, associated with the cables running between the ultra-low current (ULC) unit of the potentiostat and the EC cell. The serial resistor, R_s, acted as a current limiter, to prevent damage to the tip in case of contact with a highly conductive site on the sample surface.

Further to R_s, the circuit included a parallel resistor of R_p ~147 MΩ, to allow a finite current when the tip is off-contact and, thus, stabilise the operation of the potentiostat. Hence, the measured resistance, R_m, needs to meet the equation

$$\frac{1}{R_m} = \frac{1}{R_d + R_s^{tot}} + \frac{1}{R_p} \quad , \tag{S1}$$

which describes the total resistance of $R_d + R_s^{tot}$ in parallel with R_p. Rearrangement yields the following expression for R_m:

$$R_m = \frac{R_d + R_s^{tot}}{1 + \frac{R_d + R_s^{tot}}{R_p}} \quad . \tag{S2}$$

In the limit $R_p \gg R_d + R_s^{tot}$, one can write $R_m \approx R_d + R_s^{tot}$. That is, the current through R_p is vanishing. Thus, R_p can be neglected unless the value of $R_d \gg R_s^{tot}$ is similar to R_p. In particular, for the case of $R_d + R_s^{tot} = R_p$ one obtains $R_m = \frac{(R_d + R_s^{tot})}{2}$.

The measured data $R_m(R_d)$ and the fit result based on Eq. (S2) are shown in Fig. S5. The obtained values for the fit parameters R_s^{tot} and R_p are ~ 751.1 and ~ 147041.2 kΩ, respectively. The curve based on the linear approximation to Eq. (S2), i.e. in the limit $R_p \gg R_d + R_s^{tot}$, deviates from the fit curve for R_d values above ~5 MΩ.

For the actual c-AFM measurement, R_d can be replaced with the true tip-sample resistance R_{ts}, and the latter can be calculated from the measured resistance, R_m. By rearranging Eq. (S1), one obtains:

$$R_{ts} = \frac{R_p R_m}{R_p - R_m} - R_s^{tot} \quad . \tag{S3}$$

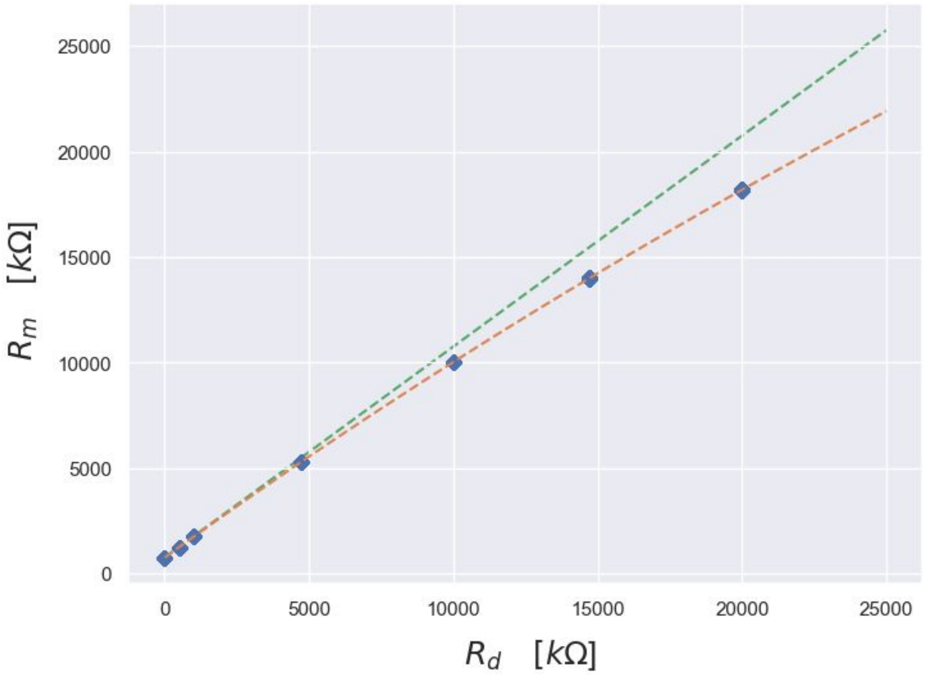

Figure S5. Plot of the resistance values, R_m, as obtained from *I-V* curves (*not shown*), for a range of dummy resistors, R_d. The regression based on Eq. (S2) yields a curve (dashed *red* line) that is in excellent agreement (coefficient of determination (COD) ~ 0.9999994) with the measured data (*blue* circles). In contrast, in the case of the linear curve (dashed *green* line) a deviation from the measured values occurs for R_d > 5 MΩ. The linear curve was calculated based on the linear approximation to Eq. (S2), i.e. as obtained in the limit $R_p \gg R_d + R_s^{tot}$.

6) Complementary example of c-AFM and friction force imaging of an NSL sample, in air

Corresponding to the self-assembly of a hexagonal close-packed (hcp) monolayer of colloidal beads in the course of the NSL procedure, a hexagonal arrangement of islands around the bead shadow area is observed. Each island acts as a junction between three adjacent hexagonal arrangements, and adjacent islands face each other in a vortex-to-vortex manner. Defects in the self-assembled shadow mask will result in deviations from the ideal pattern, such as two adjacent islands being connected to each other in the region where their vortices would be facing each other (Fig. S6a). This is a manifestation of an imperfect local packing density of the colloidal latex beads when the metal deposition was carried out, typically ranging over a meso-scale region that covers a number of beads.

As can be seen from Fig. S6, current and friction force contrasts, similar to those of Figs. 2b and 2c, respectively, were consistently observed using solid Pt type probes. The cantilever spring constant of the solid Pt probes used in this study was in the range of ~4.3 to 8.8 N/m, as determined by the thermomechanical noise method [5] in combination with measurements of force-distance curves on a stiff surface for determination of the optical lever sensitivity. With a typical setpoint value of 0.13 V, chosen to keep the applied load low while maintaining stable imaging in constant force mode, the load was in the range of ~120 to 226 nN. Operation in this force range allowed the observation of the current and friction force contrasts, and repeated imaging of the same surface area did not show signs of surface wear. Notably, repeated scanning in contact mode confirmed the structural robustness of the CuO_x islands in air, as there was no indication of the islands getting dislodged by the scanning tip.

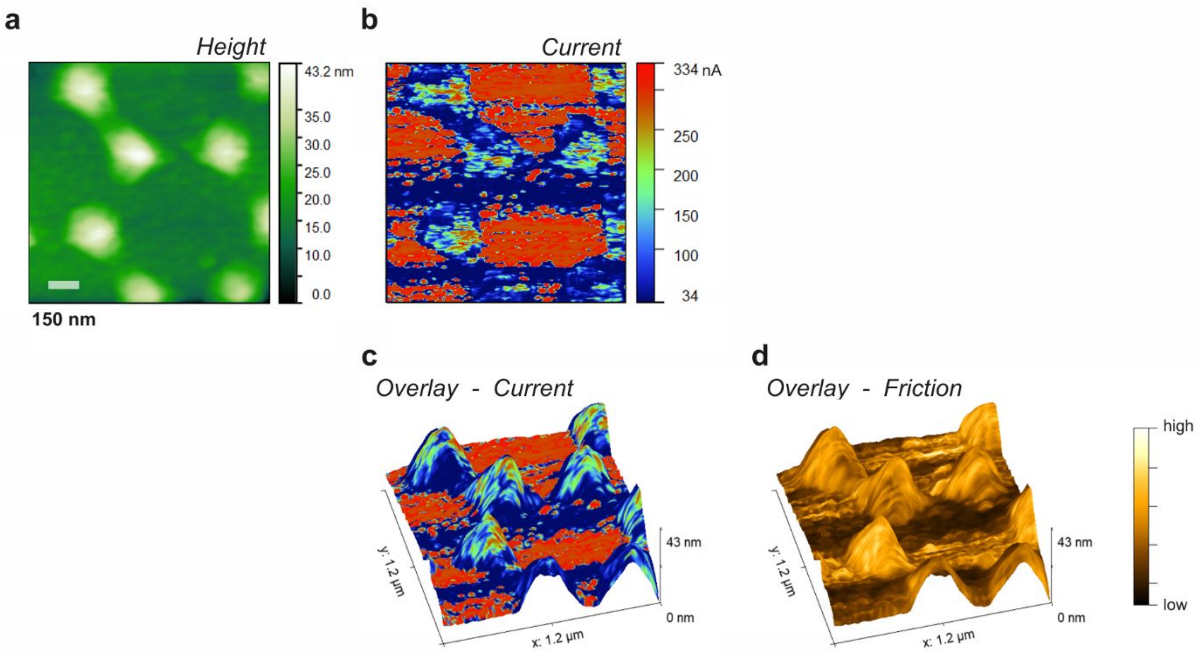

Figure S6. c-AFM imaging of an NSL-patterned Au-Cu surface, in air, using a solid Pt probe. a Height image, where the Cu islands on top of the Au film surface appear bright. **b** Current image, measured in air at E_i ~ -257 mV. Corresponding to Ei <0, the measured current was <0 but for simplicity absolute current values are given. **c** Overlay of the current signal onto a 3D representation of the height image. **d** Similarly, an overlay of the friction force signal. On average, the measured current was lower on the islands, as can be seen from b and c. In contrast, the friction force signal was larger on the islands than on the Au surface.

7) *I-V* and noise characteristics for the case of polycrystalline Au surfaces in water

For the case of *I-V* measurements on Au in air (Fig. 3c), the signal-to-noise ratio (SNR) was largely constant over the relatively narrow voltage range. Besides, reference measurements on a polycrystalline Au surface in water indicated a mild gain in SNR with stepwise increased applied potential from ~ +170 to +770 mV vs. Ag/AgCl.

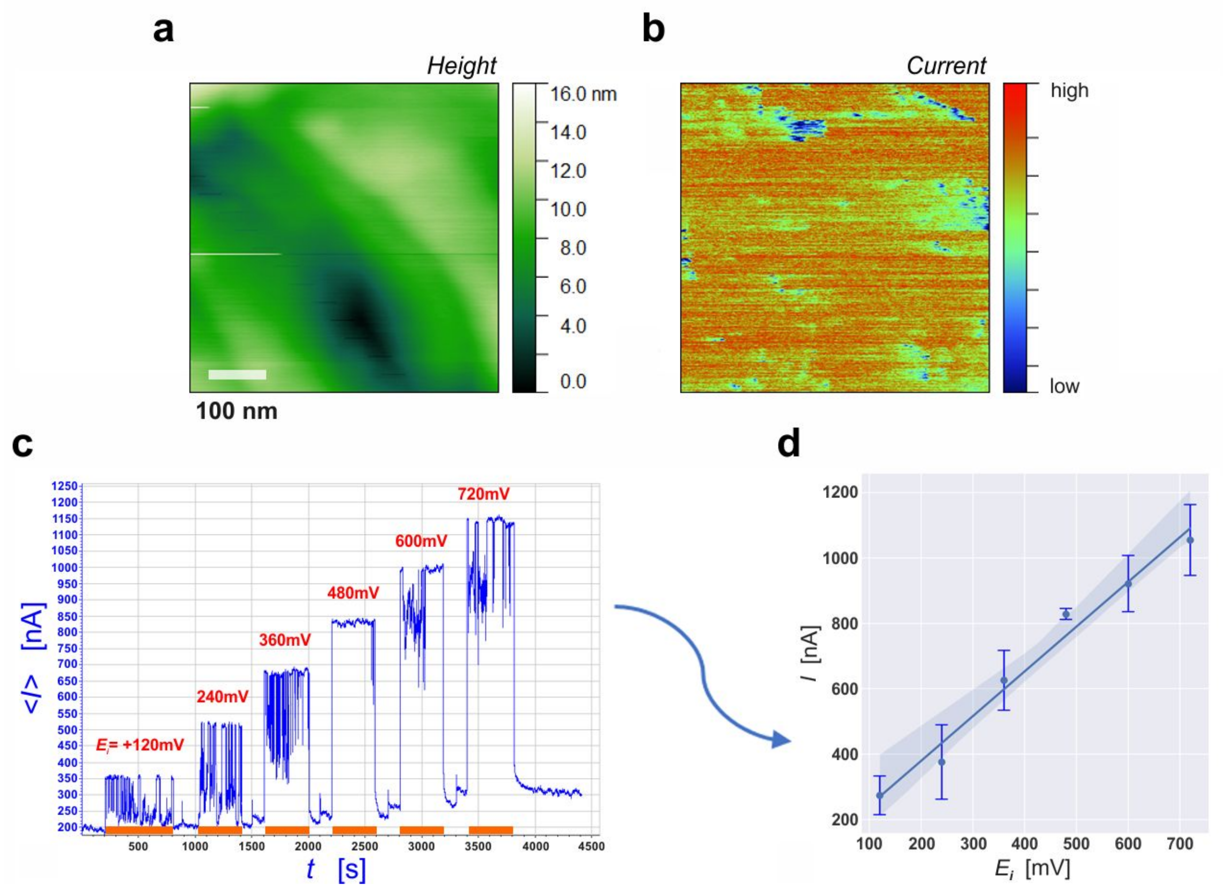

Figure S7. c-AFM imaging and stepwise variation of the potential E_i. a Height image of an Au polycrystal of ~1.7 mm thickness. **b** Current image, measured at E_i ~ +530 mV vs. Ag/AgCl. **c** Chronoamperometric (CA) trace of the current, *I*, measured while the applied potential, E_i, was increased in a stepwise manner. To evaluate the noise level, each E_i value was held for ~400 s, while the AFM tip was dwelling on the same surface spot. It should be mentioned that the range of applied voltages was relatively wide in this case, up to ~720 mV vs. Ag ($E_i = n \cdot 120$ mV, with *n*= 1 to 6). Usually, the voltages applied during c-AFM imaging were in the range between ~ -600 to +530 mV vs. Ag/AgCl. **d** Corresponding plot of the mean *I* values vs. the E_i values. Similar to the analysis of *I-V* curves, the overall resistance can be obtained from the slope of the linear fit to the measured data. Corresponding to a value of ~1.37 nA/mV, the overall resistance amounts to ~0.73 $\cdot 10^6$ V/A = 0.73 MΩ. Consultation of Eq. (S3), which accounts for the serial and parallel resistances of the front-end circuit, shows that the tip-sample resistance, R_{ts}, was close to zero, thus confirming high electric conductivities of both the TiN coated AFM tip and the Au polycrystal.

To evaluate whether the *I-V* characteristics observed in deionized water are a function of the chosen electrode configuration, we carried out control measurements in the usual three-electrode configuration (Fig. S8b) as well as in a two-electrode configuration (Fig. S8a). In the latter case, the RE cable of the potentiostat was connected to the CE cable. Throughout these measurements, the probe (here of the solid Pt type) and the EC cell (with a polycrystalline Au surface mounted) were unchanged. There was thus no evidence for significant variations in the resistance values upon changing the electrode configuration (Fig. S8).

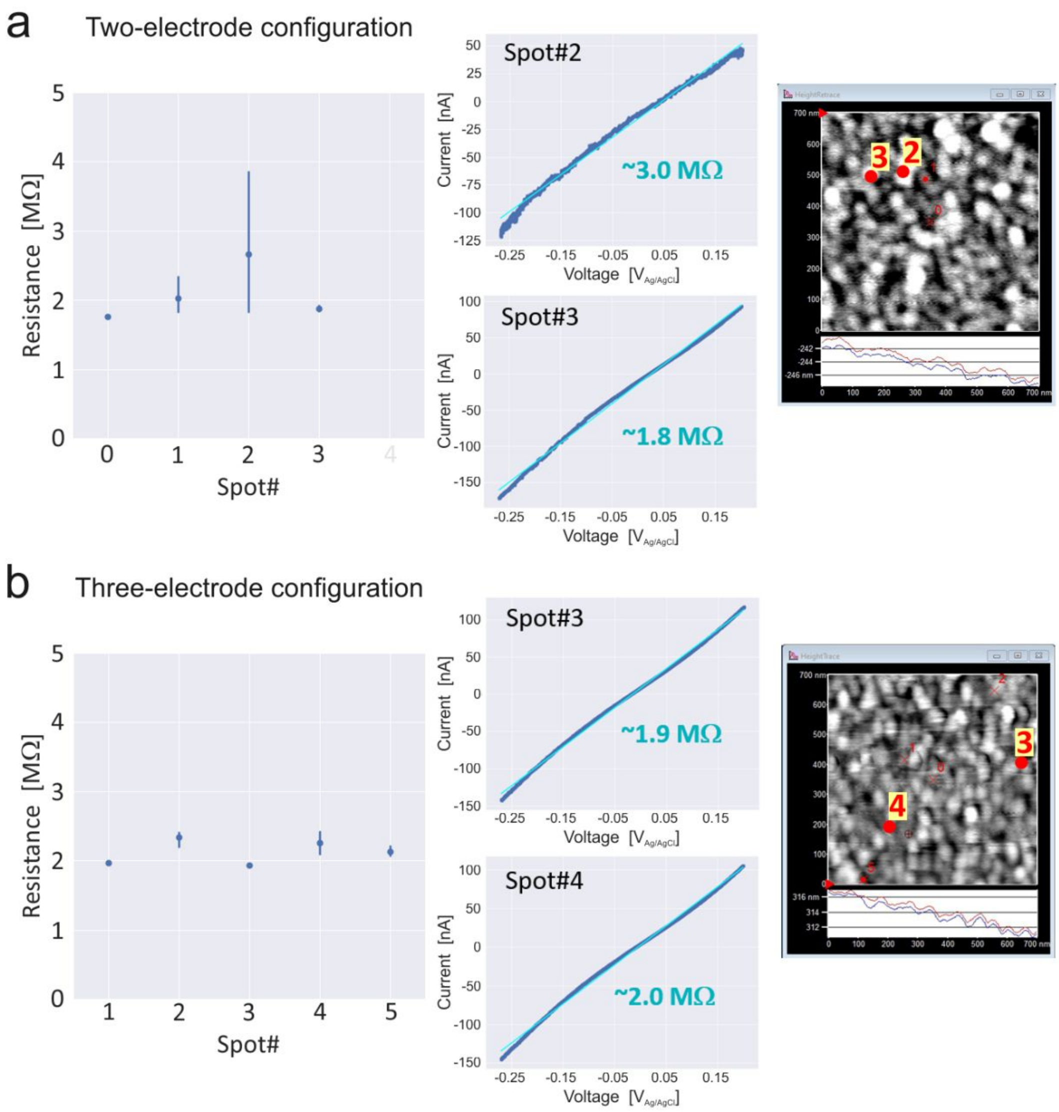

Figure S8. Two- versus three-electrode configuration, for the case of a polycrystalline Au surface in water. The measurements were carried out using a solid Pt probe. a Results obtained in the two-electrode configuration, including a plot of the mean resistance value vs. the spot number (*left* column) and 2 typical *I-V* curves (*middle* column). Height images with the spot positions marked are shown in the column on the *right*. **b** Results obtained in the three-electrode configuration.

8) c-AFM based *I-V* curve measurements on an NSL sample, in the electrolyte

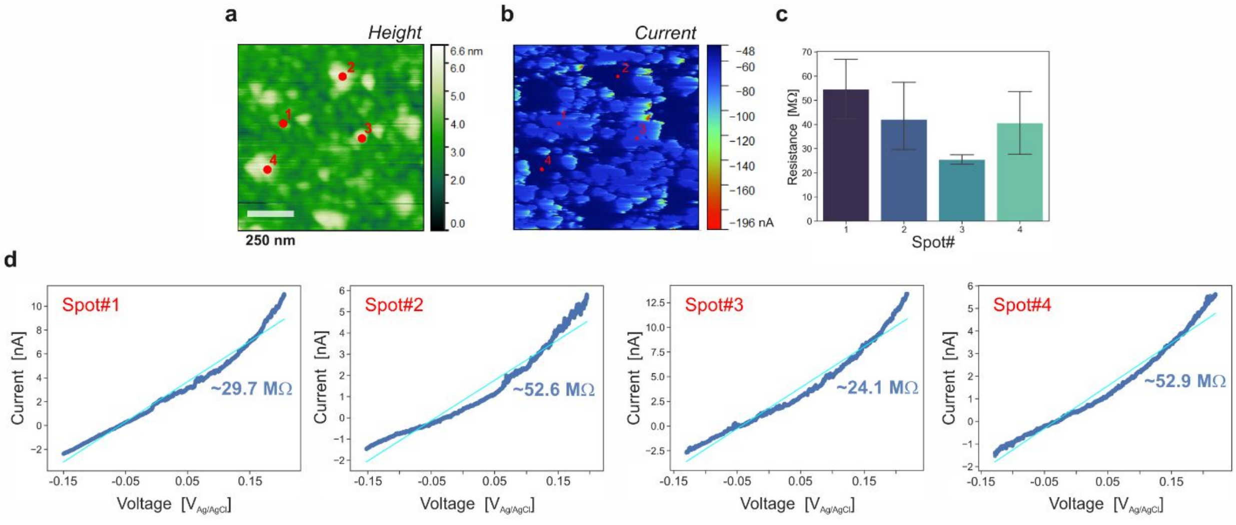

Figure S9. Results of *I-V* curve measurements on selected spots of the scan shown in Figs. 2g-i. a Height image, including markers for the selected spots. **b** Current image, measured at E_i ~ -597 mV vs. Ag/AgCl. Corresponding to *Ei* <0, the measured current was <0 but for simplicity absolute current values are given. **c** Bar plot of the measured resistance values at the four selected spots. **d** Panel with typical *I-V* curves measured at the selected spots and associated linear fit curves. The Spots #2 and #4 were located on Cu islands, whereas Spot#3 was on the Au film. Supposedly, Spot#1 was on Au but the corresponding resistance value was relatively large. This deviation may have been caused by local adsorption processes occurring subsequently to the imaging, thus increasing the tip-sample contact resistance.

Using Eq. (S3), the resistance values from the linear fits to the *I-V* curves were converted to R_{ts} values. The mean R_{ts} value associated with the curves measured on Spot#1 seemed relatively large (R_{ts} ~54.5 ± 19.4 MΩ) compared to the contrast of the current image (Fig. 2h). This deviation could have been caused by a temporarily adsorbed adlayer in the Au region of Spot#1, thus increasing the overall R_{ts} value.

The mean R_{ts} values for each condition are summarised in Table ST1. For instance, the R_{ts} values relating to the measurement in the electrolyte were $R_{ts,\mathrm{Cu}}$ ~57.0 ± 43.6 MΩ (CuO_x islands) and $R_{ts,\mathrm{Au}}$ ~30.0 ± 4.2 MΩ (Au). Considering the measurement uncertainties, the $R_{ts,\mathrm{Cu}}$ values obtained in air and in water were similar (Table ST1). A significant increase in the R_{ts} values measured on Au and CuO_x islands occurred when measuring in the electrolyte.

Table ST1. Overview of the tip-sample contact resistance, R_{ts}, values from *I-V* curve measurements in air, water and the electrolyte. The uncertainties given are the single standard deviation.

Region of the NSL pattern	R_{ts} [MΩ] in air	R_{ts} [MΩ] in water	R_{ts} [MΩ] in the electrolyte
Au	0.14 ± 0.01	0.90 ± 0.60	30.0 ± 4.2
CuO_x	10.2 ± 3.7	4.0 ± 8.6	57.0 ± 43.6

Further to random curve-to-curve fluctuations, conceivable sources of uncertainty in the determined R_{ts} values are limitations associated with the relatively small size of the CE, i.e. the AFM tip, or variations in the distance of the QRE from the sample/electrolyte/tip junction.

In general, the current scales with the size of the contact area, which is a function of the load F_c applied by the AFM cantilever. Assuming Hertzian contact mechanics, the contact radius a is given by $a^3 = (r_t / K)F_c$.. Here, r_t is the radius of curvature of the tip apex and K denotes the reduced modulus (calculated from the tip and sample materials' Young's modulus and Poisson's ratio values). In the case of an ohmic contact, the spreading resistance is given by $R_{\mathrm{Spreading}} = \rho /(2a)$, with the mean resistivity ρ of the tip and sample materials [6]. In the case of an electron mean free path L (~37 nm for Au at 300 K) larger than the contact radius, the contact resistance (in vacuum) can be described by the Sharvin equation $R_{\mathrm{Sharvin}} = 4L\rho / (3\pi a^2)$ [6]. While these equations illustrate the relationship between load, contact radius and contact resistance, it seems unlikely that they will give accurate values in the case of a nanoscale contact involving a layer of the electrolyte. With the aim to estimate the resistance variation with the load, however, in a first approximation we use the equation for the spreading resistance and obtain a ratio of $R_{\mathrm{Spreading,1}}/ R_{\mathrm{Spreading,2}}= (F_{c2}/F_{c1})^{1/3}= (226/120)^{1/3}$ ~1.23. Here, we were considering that the load was in the range of ~120 to 226 nN in the case of the solid Pt probes used.

It is interesting to note that a variation of $(R_{\mathrm{Spreading,1}} - R_{\mathrm{Spreading,2}}) / R_{\mathrm{Spreading,2}}$ ~0.23 is relatively small compared to the difference in resistance values measured on Au and CuO_x islands (looking at Table ST1 in SI Sec. 8, the relative differences in resistance, $(R_{\mathrm{CuOx}}-R_{\mathrm{Au}})/R_{\mathrm{Au}}$, amount to ~71.9, 3.4 and 0.9 in air, water and electrolyte, respectively).

9) *B*-coefficient of the Jones-Dole relationship between electrolyte concentration and viscosity

The effect of chao- or kosmotropic ions on the relative viscosity is reflected by the *B* coefficient of the Jones-Dole relationship, an empirical expression for the electrolyte viscosity as a function of its molar concentration [7], [8], [9]. As laid out in the review article by Jenkins and Marcus [7], the relative viscosity η/η_0 can be written as

$$\frac{\eta}{\eta_0} = 1 + Ac^{1/2} + Bc + Dc^2 \quad , \qquad \text{(S4)}$$

with the molar concentration c of the electrolyte, the electrolyte viscosity η and the solvent viscosity η_0. The coefficients A, B and D depend on the solute, solvent and temperature. The term in c^2 tends to be neglectable for low concentrations $c <$ 0.5 mol/dm^3. By plotting experimental viscosity data, obtained for a low-concentration electrolyte, in the form

$$\left[\frac{\eta}{\eta_0} - 1\right] c^{1/2} = A + Bc^{1/2} \quad , \qquad \text{(S5)}$$

the coefficients A and B can be determined from the intercept and the slope of the linear curve, respectively. While A (in dm$^{3/2}$ mol$^{-1/2}$) depends on the ion-ion interaction, B (in dm^3 mol^{-1}) depends on ion-solvent interactions and is related to the ion volumes. For kosmotropes (i.e., structure-making ions), such as Li^+ and Na^+, the B coefficient is positive, whereas for chaotropes (i.e., structure-breaking ions), such as Cs^+, it is negative [7], [8]. For K^+ in aqueous solutions, a small negative B value is given in Ref. [7], thus suggesting weak chaotropic behaviour, if any.

Looking at anions present in the bicarbonate electrolyte, carbonate ions were reported to show kosmotropic behaviour [8].

10) Copper island height variations upon exposure to water and 100 mM $KHCO_3$ aq. electrolyte

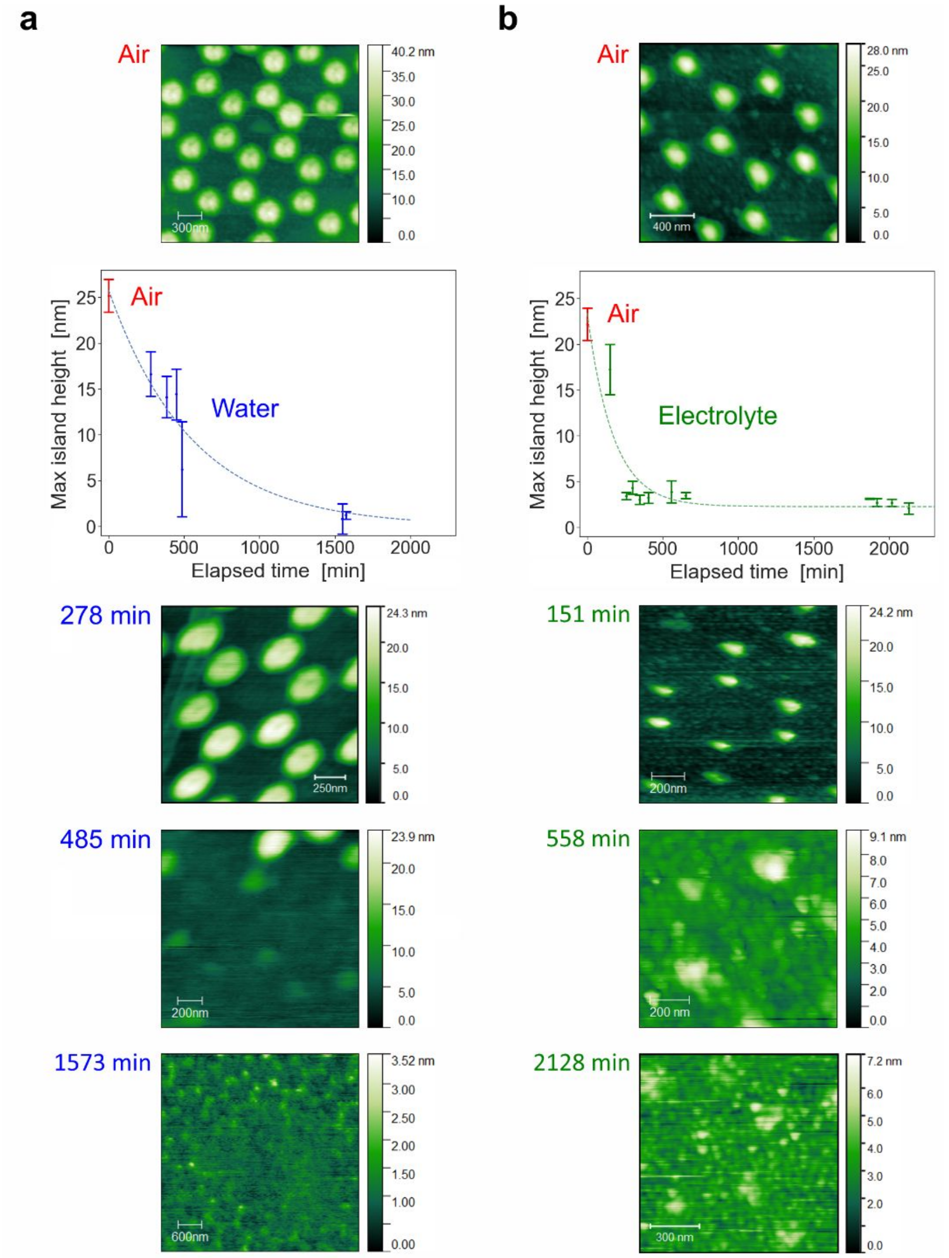

Figure S10. Decrease of the copper island height upon exposure to aqueous media. a The case of ultrapure water. **b** The case of a 100 mM $KHCO_3$ aqueous electrolyte. The height values given are the mean values of the maximum Cu island height (obtained for a number of islands within the scanned area), and the error bars are given by 1x the corresponding standard deviation. The values for $t = 0$, i.e. at the starting point of the time series, were obtained from height images measured in air (points in *red*) prior to filling the cell with the liquid. The dashed lines resulted from fits, using of a single exponential decay function, and suggested decay times in the order of 10^2 min.

In situ AFM imaging further allows concurrent tracking of interfacial corrosion processes which can strongly affect the electrocatalyst's performance. For both exposure to water and electrolyte, the CuO_x island height was found to gradually decrease over time. Figures 2g-i exemplify CuO_x islands upon exposure to the electrolyte, with their height significantly reduced from an initial value of ~22.2 ± 1.8 nm

(as measured in air, see Fig. S10b) to ~3.2 ± 0.6 nm (after ~403 min of exposure to the electrolyte). The gradual decrease in island height (Fig. S10) and, thus, volume, indicates a dissolution process in the presence of aqueous electrolytes. The *in situ* imaging of the morphology, ideally combined with current imaging, opens up the possibility of analysing, in real-space, the relationship between interfacial corrosion and local electric conductivity, thus gaining mechanistic insights into defects and local corrosion domain formation.

Following the bulk Cu Pourbaix diagram, oxidizing conditions in the acidic regime (pH < 6) favour copper oxide dissolution to aqueous Cu^{2+} [10]. Based on pK_S values for solvation of metal oxides [2], [11], the solubility of CuO (pK_S -23.5) is expected to be much lower than that of Cu_2O (pK_S -9.0), causing slower dissolution of CuO_x islands that are covered by CuO rather than Cu_2O (Fig. S3).

11) Description of the relative contribution of potentially occurring leak currents

As non-zero leak currents can impair both the spatial resolution and the current sensitivity, *in situ* electrochemical AFM measurements give rise to the probe fabrication challenge of depositing an insulating top layer onto the electrically conductive probe, while maintaining conductivity at the tip apex [12], [13]. Yet, our *in situ* c-AFM results, as demonstrated in ultrapure water and in 100 mM $KHCO_3$ aqueous electrolyte, show that imaging using conductive probes with no further insulating sheath is feasible and yields valuable contrasts.

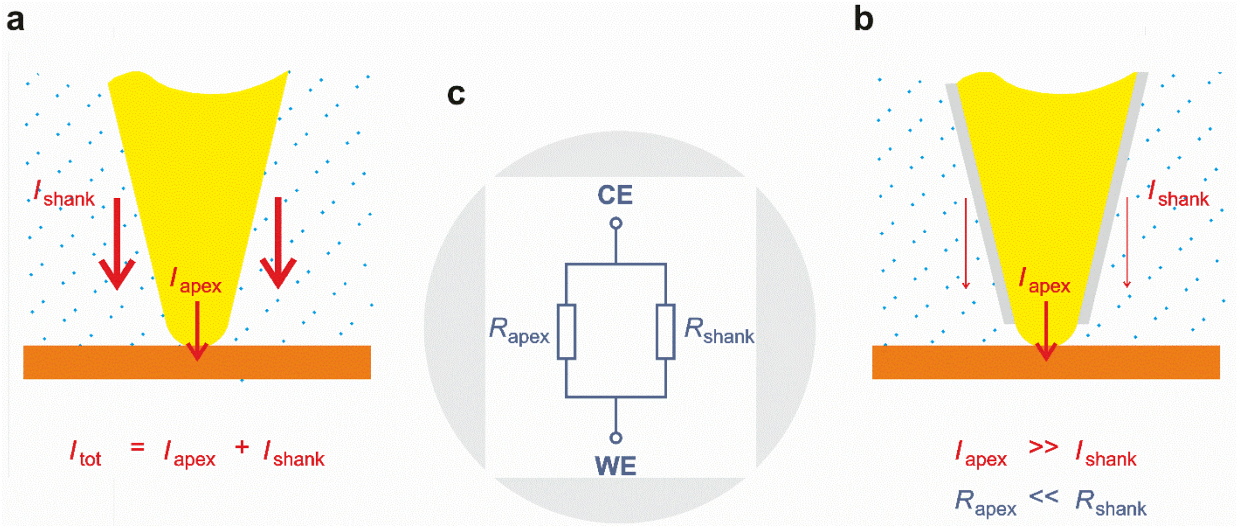

Figure S11. Schematic representation of an electrically conductive tip. It can be thought of a tip apex and a tip shank component. **a** In the case of a bare but conductive tip surface, a current I_{shank} can occur when operating in an electrolyte. **b** In the case of an insulating layer on top of the tip shank, the current component I_{shank} will be significantly reduced or even eliminated. **c** Equivalent circuit depicting the tip as a parallel configuration of the resistors R_{apex} and R_{shank}, corresponding to the respective surface-liquid resistances. While successful electric insulation of the tip shank will result in R_{shank} >> R_{apex}, in the case of a poor or missing tip shank insulation the resistance R_{shank} will be a function of the electrolyte type and concentration.

12) Grain size distribution of the polycrystalline gold film

The measured grain size distribution was in the range between ~2 and 38 nm, as expressed in terms of the radius, r_{eq}, of an equivalent disc with the same area as the projected area of the grain. It should be however mentioned that these experimentally determined corner values of the grain size distribution are likely to be larger than the true radii, due to the tip convolution effect associated with the finite size of the AFM tip. In fact, a grain diameter of 2x 38 nm would be larger than the nominal thickness (~50 nm, as specified by the manufacturer) of the Au film. Assuming that the measured grain width, w', can be written as $w' = w + 2R_t$, the tip radius is $R_t = \left(w' - w\right)/2$, where w denotes the true grain width. Hence, a plausible value of R_t ~13 nm is obtained if w_{m}' ~76 nm and w_{m} ~50 nm.

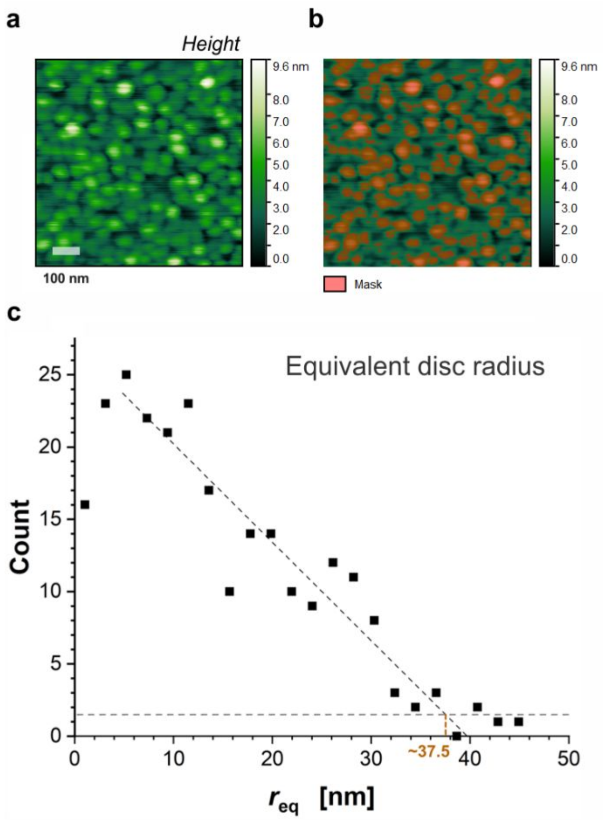

Figure S12. Grain size analysis of a gold film surface. a Height image, as measured in 5 mM K_2CO_3 aqueous electrolyte with dissolved CO_2 and using a solid Pt probe. **b** Segmentation of the height image by the Watershed method [14], [15], with the detected grains marked by a mask. **c** Grain size distribution, in terms of the equivalent disc radius, r_{eq}. The largest r_{eq} value is ~37.5 nm, approximately, as given by the intersection of the two linear segments (*dashed lines*).

13) Curvature and mean current of individual gold film grains

By way of parabolic fits to the height profiles *z*(*x*), the grain shape can be described effectively in terms of the three fit parameters *a*, *b*, and *c* of the zeroth, first and second order term, respectively. With the parameter *c* as a measure for the grain curvature *z*''(*x*)= 2*c*, a facile and yet effective parameterization of the grain shape was accomplished, as the parabolic function allowed in most cases for very good agreement between the fit curve and the measured grain profile (Fig. S13a).

Figure S13d would suggest that grains with higher peaks are more curved (Pearson correlation coefficient, PCC ~0.576). However, a strict relationship between grain height and size cannot be expected for grain-on-grain configurations, which are likely to occur for grains with a diameter (Fig. S12) below the Au film thickness of ~50 nm.

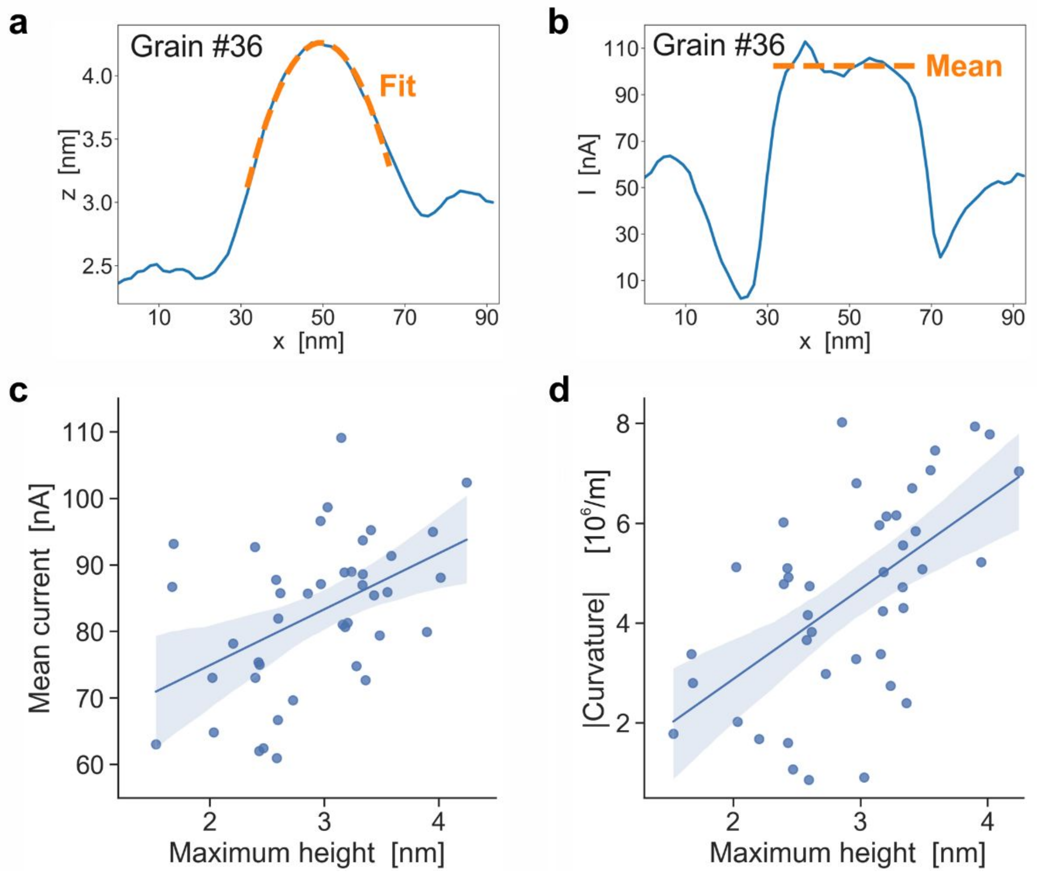

Figure S13. Shape and current of individual gold film grains. a Cross-sectional height profile of grain #36, as marked in Fig. 4a. **b** Cross-sectional current profile of grain #36. **c** Plot of the mean current of individual grains *versus* their maximum height, with the linear trend corresponding to a Pearson correlation coefficient, PCC, of ~0.470. **d** Plot of the curvature of individual grains *versus* their maximum height (Pearson correlation coefficient, PCC ~0.576).

14) Single observation of a reverted c-AFM current contrast between granular and intergranular regions

Considering that the electric current tends to scale with the contact area, as known from c-AFM experiments in vacuum [6], one might expect increased electric currents measured on the valley-like, i.e. concave, morphology of intergranular regions, where the tip-sample contact area is likely to be larger than on a flat or convex area (Fig. S16a). Nonetheless, it should be mentioned that such contact area variation is also a function of the ratio between tip size and feature size, as AFM tips with a relatively large apex cannot reach into narrow valleys (Figs. S16b,c). Only in one case the electric current appeared higher in intergranular regions than on the Au grains (Fig. S14). The bent shape of the AFM tip, as evidenced by retrospective SEM imaging, most likely caused an increased total contact area including contact points between the tip shank and the intergranular Au grain sidewalls.

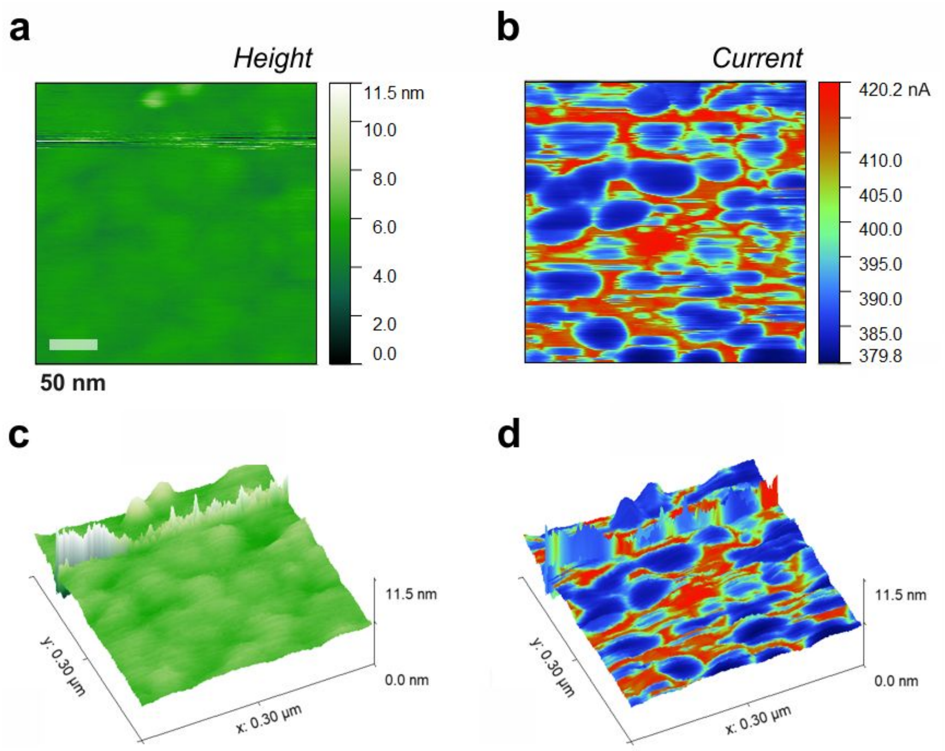

Figure S14. c-AFM images of a polycrystalline gold surface in ultrapure water, using a solid Pt probe which happened to be bent near its foremost end. a Height image of the polycrystalline gold surface. **b** Current image, measured at E_i ~ -200 mV vs. Ag/AgCl. Corresponding to E_i <0, the measured current was <0 but for simplicity absolute current values are given. **c** 3D representation of the height image. **d** Overlay of the current contrast onto the height image 3D representation. Gold grains appear elevated relative to the surrounding intergranular regions, where a locally increased current was measured in this special case.

15) Locally-reduced current associated with a single grain

The tip-sample current dropped when scanning across a certain grain, as can be seen from its dark appearance in the current image of Figs. S15b,d. The friction force contrast of this grain was distinct too, as the measured friction was lower than on the surrounding grains. Hence, exemplary non-conductive regions were detected that would correspond to a (temporary) area of vanishing electron transfer and, thus, hardly contribute to the electrocatalyst's active surface area.

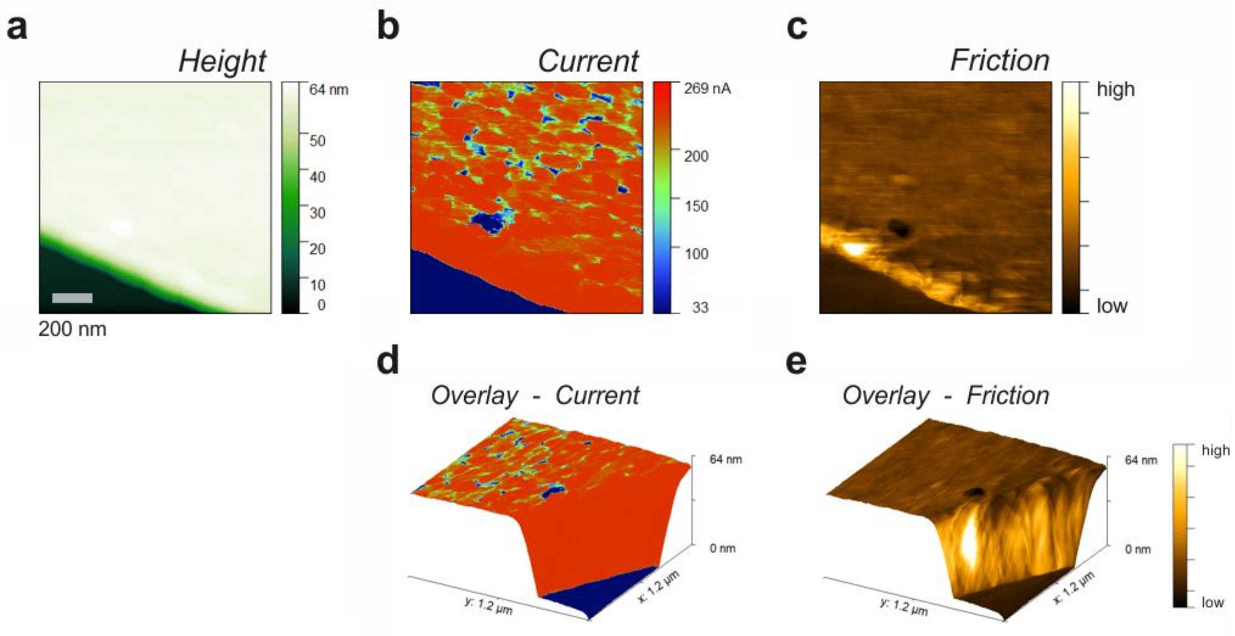

Figure S15. c-AFM imaging in air, using a solid Pt probe, of two Au microelectrodes separated by a gap. a Height image of the area around the gap. The gap width and scan width were ~750 nm and 2.0 µm, respectively. **b** Current image, measured in air at E_i ~ -147 mV. Corresponding to E_i <0, the measured current was <0 but for simplicity absolute current values are given. Note the stark contrast between the Si wafer surface (*dark*) and the upper right Au electrode (*bright*). Near the electrode edge a grain can be seen that appears much darker than the surrounding Au grains, i.e. the tip-sample current measured on this grain was much lower. **c** Friction force image measured in a subsequent scan (scan width 1.2 µm). The same grain that appears distinct in the current contrast also shows a clear friction force contrast, i.e. the friction force signal was lower than on the surrounding grains. **d** Overlay of the current signal. **e** Overlay of the friction force signal.

16) Effect of the finite tip size on the AFM imaging of a polycrystalline morphology

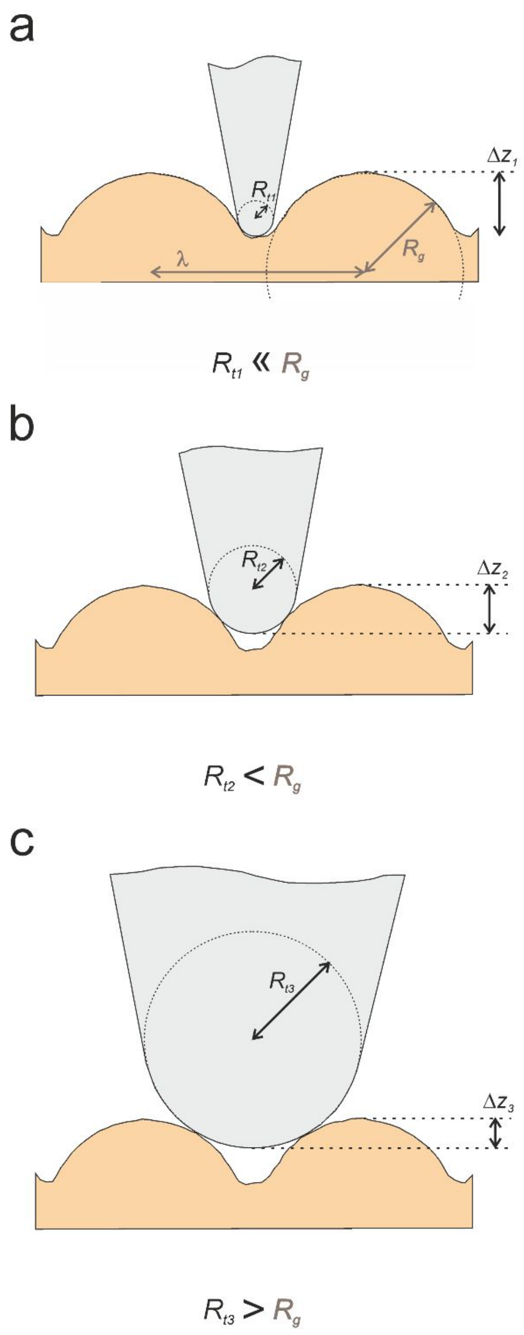

Figure S16. AFM tip size *versus* the grain size of a polycrystalline surface. a The case of a sharp tip with a radius R_t much smaller than the typical grain radius R_g (R_{t1} = R_g). **b** The case of a less sharp tip ($R_{t2} < R_g$). **c** The case of a blunt tip ($R_{t3} > R_g$). The characteristic grain-to-grain distance and the measured height variation are denoted as λ and Δz, respectively.

17) Correlative analysis of height, current and friction force images of a polycrystalline Au electrocatalyst surface in water

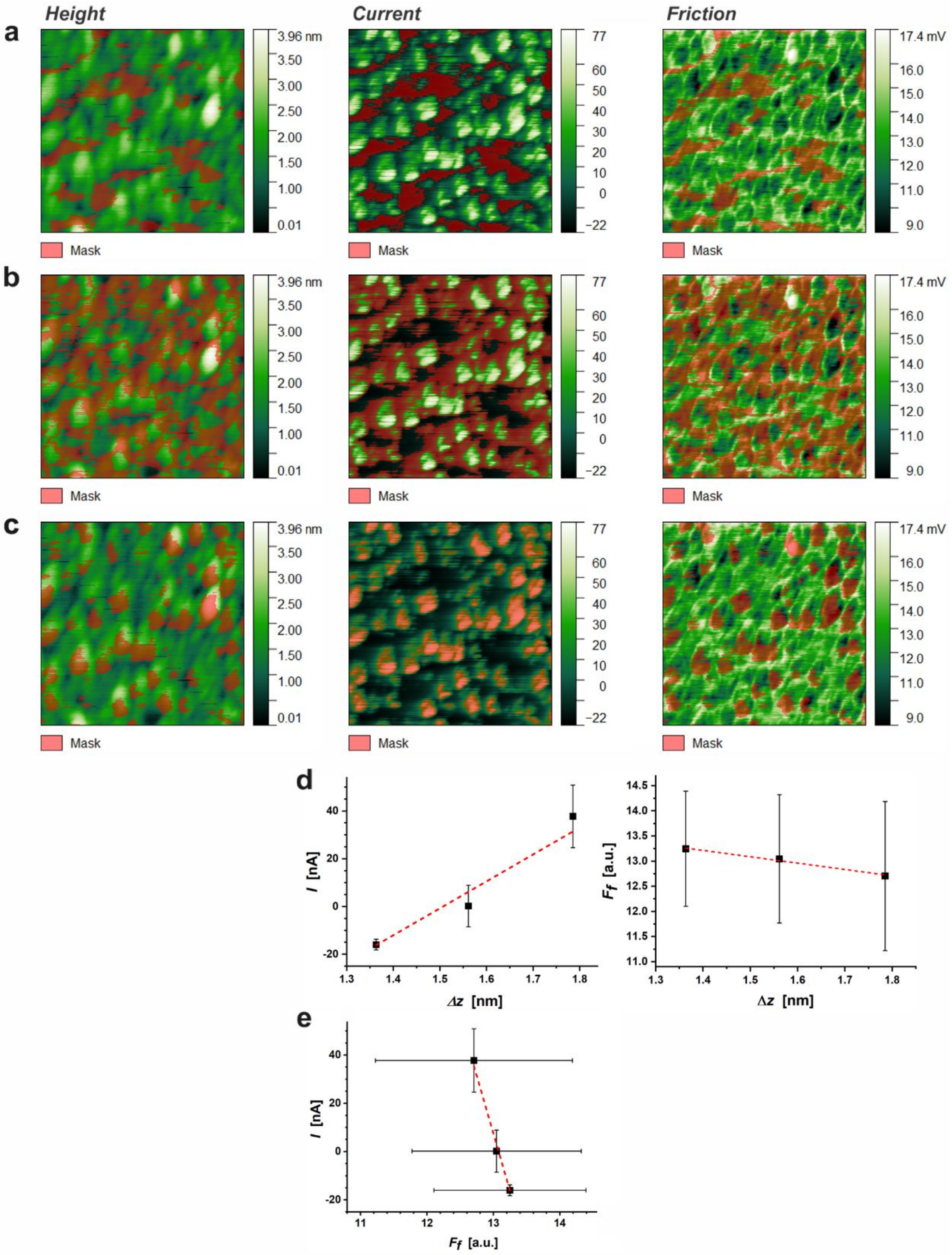

Figure S17. Correlative analysis of a polycrystalline Au electrocatalyst surface imaged in water. a Areas of low current (range 0 to 10%) were marked for analysis. Using the mask derived from the current image (*middle* column), the same areas were selected in the corresponding height (column to the *left*) and friction force (column to the *right*) images. **b** Same as depicted in (a) but for the current range 10 to 42%. **c** Same as depicted in (a) but for the current range 42 to 100%. **d** Plots of the current, I, and friction force, F_f, versus the height, Δz. **e** Plot of I vs. F_f.

By partitioning the current range of Fig. 4e into three consecutive intervals, potential correlations between these quantities can be identified more readily. The partitioning of the current image was applied to the height and friction force images (Figs. S17a-c), and the corresponding mean values from each partition were plotted. Indeed, the mean current was found to increase with the mean height of the partition (Fig. S17d).

Nonetheless, it should be borne in mind that any potentially occurring spatial property variations directly associated with the morphology of the polycrystalline Au surface and its intergranular regions would be superimposed to property variations originating from interfacial ordering of electrolyte species.

18) Electron energy scheme for the interfacial electron transfer from a metal electrode

Alongside the applied voltage and the metal work function, the interfacial electron transfer (IET) from the working electrode surface to the electron acceptor is a function of the energy levels of the accepting species in the solution, In these terms, the AFM tip can be thought of as an electron acceptor (while the electrocatalyst is at a negative potential), mimicking an oxidized species in the electrolyte.

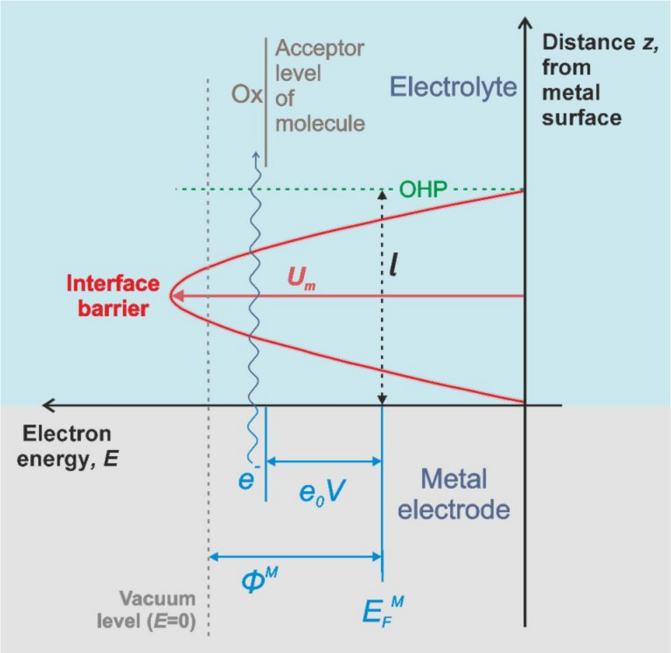

Figure S18. Electron energy scheme for the IET from a metal electrode. The electron transfer occurs to an acceptor molecule in the electrolyte, across an energy barrier between the metal surface and the outer Helmholtz plane (OHP), i.e. the nearest plane where hydrated ions or molecules reside. The width and maximum height of the energy barrier are given by l and U_m, respectively. The metal electrode is characterised by its Fermi energy E_F^M and work function Φ^M. The energy shift caused by the applied voltage V is e_0V, with the elementary charge e_0. Adapted with permission from Ref. [16] (T. Liu *et al.* 2019 *J. Phys. Chem. C* 123, 28319–28326). Copyright 2019 American Chemical Society.

19) c-AFM and friction force contrasts of a polycrystalline Au surface, in air and in a mixed electrolyte

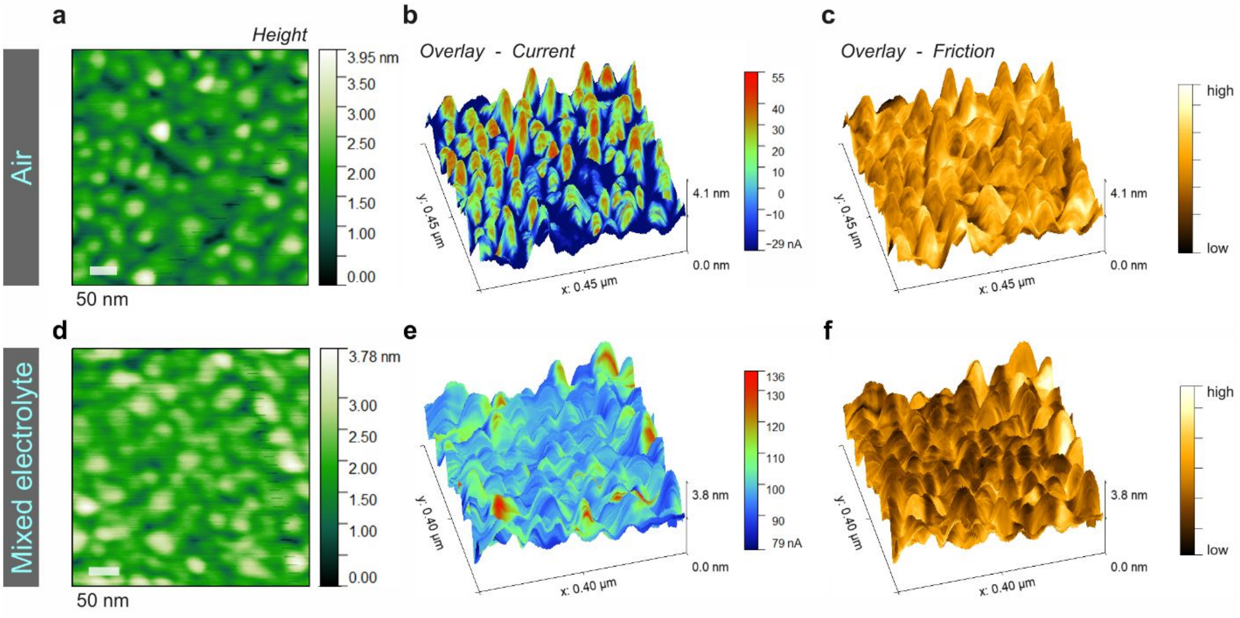

Figure S19. c-AFM of a polycrystalline Au surface, using a TiN coated cantilever. a Height image, in air, measured using a TiN coated tip. **b** Overlay of the current image, measured at E_i ~+270 mV vs. Ag/AgCl, onto a 3D representation of the height image. **c** Overlay of the friction force signal. **d** Height image, as measured in a mixed aq. electrolyte of CO_2-saturated 100 mM $KHCO_3$ with 50 mM KCl and using the same TiN coated tip. **e** Overlay of the current image, measured at E_i ~ +370 mV vs. Ag/AgCl. **f** Overlay of the friction force signal.

20) Electrically blocking patch on top of a Au polycrystalline film

The local reaction rate is likely to be current-limited or even vanishing in the region of the top layer which exhibits a drop in the spatially-resolved electric current. The overlay of Fig. S20b illustrates the current variations associated with a patch of ~11 nm thickness (Fig. S20a), which were relatively large compared to the variations observed on the surrounding polycrystalline Au surface. The corresponding friction force overlay (Fig. S20c) shows higher friction on the patch than on the nearby Au surface. Such electrode areas, covered by poorly conductive layers, invoke poor local catalytic activity due to locally suppressed electron flow to oxidized species. This finding demonstrates that, in contrast to global measurement techniques, our approach allows locally-resolved detection and elucidation of individual areas prone to being electrocatalytically passive.

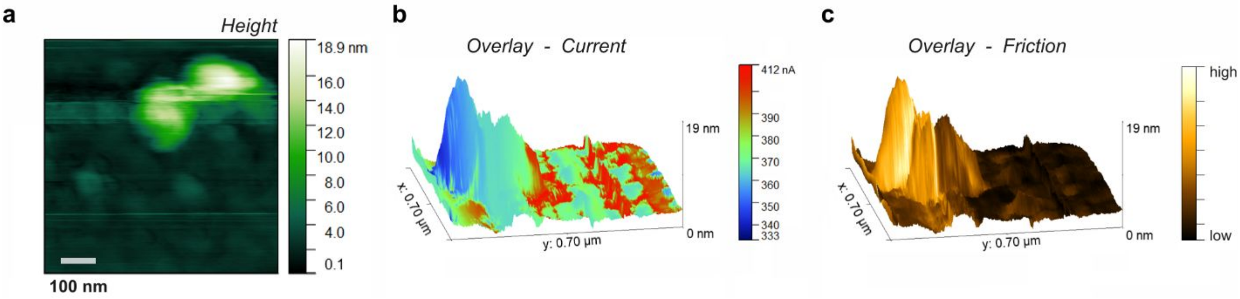

Figure S20. *In situ* current and friction force contrasts of a blocking patch, as measured in water and using a solid Pt tip. a Height image showing a patch on top of a polycrystalline Au surface. **b** Overlay of the current image, measured at E_i ~ -200 mV vs. Ag/AgCl. Corresponding to Ei <0, the measured current was <0 but for simplicity absolute current values are given. **c** Overlay of the friction force image.

Supplementary references

[1] Hans M, Mathews S, Mücklich F and Solioz M 2015 Physicochemical properties of copper important for its antibacterial activity and development of a unified model *Biointerphases* **11** 18902

[2] Mudunkotuwa I A, Pettibone J M and Grassian V H 2012 Environmental Implications of Nanoparticle Aging in the Processing and Fate of Copper-Based Nanomaterials *Environ. Sci. Technol.* **46** 7001–10

[3] Barten D, Kleijn J M, Duval J, Leeuwen H P v., Lyklema J and Cohen Stuart M A 2003 Double Layer of a Gold Electrode Probed by AFM Force Measurements *Langmuir* **19** 1133–9

[4] Dedeloudis C, Fransaer J and Celis J-P 2000 Surface Force Measurements at a Copper Electrode/Electrolyte Interface *J. Phys. Chem. B* **104** 2060–6

[5] Butt H-J and Jaschke M 1995 Calculation of thermal noise in atomic force microscopy *Nanotechnology* **6** 1–7

[6] Lantz M A, O'Shea S J and Welland M E 1997 Simultaneous force and conduction measurements in atomic force microscopy *Phys. Rev. B* **56** 15345–52

[7] Jenkins H D B and Marcus Y 1995 Viscosity B-Coefficients of Ions in Solution *Chem. Rev.* **95** 2695–724

[8] dos Santos A P, Diehl A and Levin Y 2010 Surface Tensions, Surface Potentials, and the Hofmeister Series of Electrolyte Solutions *Langmuir* **26** 10778–83

[9] Jones G and Dole M 1929 The viscosity of aqueous solutions of strong electrolytes with special reference to barium chloride *J. Am. Chem. Soc.* **51** 2950–64

[10] Osseo-Asake K and Mishra K K 1996 Solution chemical constraints in the chemical-mechanical polishing of copper: Aqueous stability diagrams for the Cu-H2O and Cu-NH3-H2O systems *J. Electron. Mater.* **25** 1599–607

[11] Feitknecht W and Schindler P 1963 Solubility constants of metal oxides, metal hydroxides and metal hydroxide salts in aqueous solution *Pure Appl. Chem.* **6** 125–206

[12] Pobelov I V, Mohos M, Yoshida K, Kolivoska V, Avdic A, Lugstein A, Bertagnolli E, Leonhardt K, Denuault G, Gollas B and Wandlowski T 2013 Electrochemical current-sensing atomic force microscopy in conductive solutions *Nanotechnology* **24** 115501

[13] Noh J H, Nikiforov M, Kalinin S V, Vertegel A A and Rack P D 2010 Nanofabrication of insulated scanning probes for electromechanical imaging in liquid solutions *Nanotechnology* **21** 365302

[14] Klapetek P, Valtr M, Nečas D, Salyk O and Dzik P 2011 Atomic force microscopy analysis of nanoparticles in non-ideal conditions *Nanoscale Res. Lett.* **6** 514

[15] Vincent L and Soille P 1991 Watersheds in digital spaces: an efficient algorithm based on immersion simulations *IEEE Trans. Pattern Anal. Mach. Intell.* **13** 583–98

[16] Liu T, Xi C, Dong C, Cheng C, Qin J, Hu S, Liu H and Du X-W 2019 Improving Interfacial Electron Transfer via Tuning Work Function of Electrodes for Electrocatalysis: From Theory to Experiment *J. Phys. Chem. C* **123** 28319–26
